# Circulating sphingosine-1-phosphate depletion is associated with endothelial activation and altered brain-endothelial S1P pathway expression in ischemic stroke

**DOI:** 10.1186/s12987-026-00828-z

**Published:** 2026-06-12

**Authors:** Lisa T. Porschen, Frank Matthes, Hana Matuskova, Lino Braadt, Gabor C. Petzold, Arne G. Lindgren, Anja Meissner

**Affiliations:** 1 Department of Experimental Medical Science, Lund University, Lund, 221 84 Sweden; 2 Wallenberg Centre for Molecular Medicine, Lund University, Lund, 221 84 Sweden; 3 Division of Physiology & Vascular Biology, Institute of Theoretical Medicine, Faculty of Medicine, University of Augsburg, Universitätsstr. 2, 86159 Augsburg, Germany; 4 Department of Vascular Neurology, University Hospital Bonn, 53127 Bonn, Germany; 5 German Center for Neurodegenerative Diseases (DZNE), 53127 Bonn, Germany; 6 Department of Neurology and Clinical Neurophysiology, University Hospital Augsburg, Augsburg, Germany; 7 Department of Clinical Sciences Lund, Neurology, Lund University, Lund, Sweden; 8 Department of Neurology, Rehabilitation Medicine, Memory Disorders and Geriatrics, Skåne University Hospital, Lund, Sweden

**Keywords:** Sphingosine-1-phosphate (S1P), Ischemic stroke, Endothelial dysfunction, Blood-brain barrier, Biomarker

## Abstract

**Background:**

Ischemic stroke remains a leading cause of disability and mortality worldwide, with limited acute therapeutic options. Sphingosine-1-phosphate (S1P) is a bioactive lipid that regulates endothelial function, vascular integrity, and immune responses, and reduced circulating S1P levels have been reported in ischemic stroke. Whether plasma S1P depletion parallels alterations in brain-endothelial S1P metabolism, receptor expression, and endothelial activation, however, remains unclear. Here, we characterized circulating S1P levels together with stroke-associated changes in brain-endothelial S1P pathway expression, markers related to endothelial activation, and blood-brain barrier (BBB) integrity.

**Methods:**

We quantified plasma S1P concentrations in patients with acute ischemic stroke (*n* = 50) and age- and sex-matched controls (*n* = 47), with follow-up assessments at 90 days. Complementary experimental stroke studies were performed using transient and permanent middle cerebral artery occlusion (MCAo) in wild-type mice and in endothelial-specific RiboTag mice (Cdh5^Cre-ER(T)) that enable selective isolation of endothelial mRNA. In parallel, human brain microvascular endothelial cells were exposed to oxygen-glucose deprivation in vitro. Endothelial activation-related markers, expression of S1P-metabolizing enzymes and S1P receptors, BBB integrity, and circulating P-selectin levels were assessed by qPCR, Western blotting, immunohistochemistry, and ELISA-based approaches.

**Results:**

Plasma S1P levels were significantly reduced in patients with acute ischemic stroke compared with controls and recovered at follow-up, consistent with findings in experimental stroke. Endothelial-specific transcriptomic profiling revealed reduced expression of sphingosine kinases, S1P-degrading enzymes, and S1P receptors (*S1pr1, S1pr3, and S1pr4*) in the ischemic brain endothelium. Lower vascular S1PR1 protein expression was associated with increased BBB disruption, and sphingosine kinase 2 protein abundance was reduced in small cerebral vessel endothelial cells of the lesioned compared to the contralateral hemisphere. These alterations were accompanied by acute changes in endothelial barrier- and activation-related markers, together with model-dependent changes in plasma P-selectin in mice. In patients, plasma P-selectin levels were not elevated acutely but showed an inverse association with plasma S1P concentrations.

**Conclusions:**

Ischemic stroke associates with acute plasma S1P depletion that parallels altered brain-endothelial S1P pathway expression, signs of endothelial activation, and BBB disruption. These findings support plasma S1P as a candidate circulating marker associated with cerebrovascular injury after stroke.

**Graphical Abstract:**

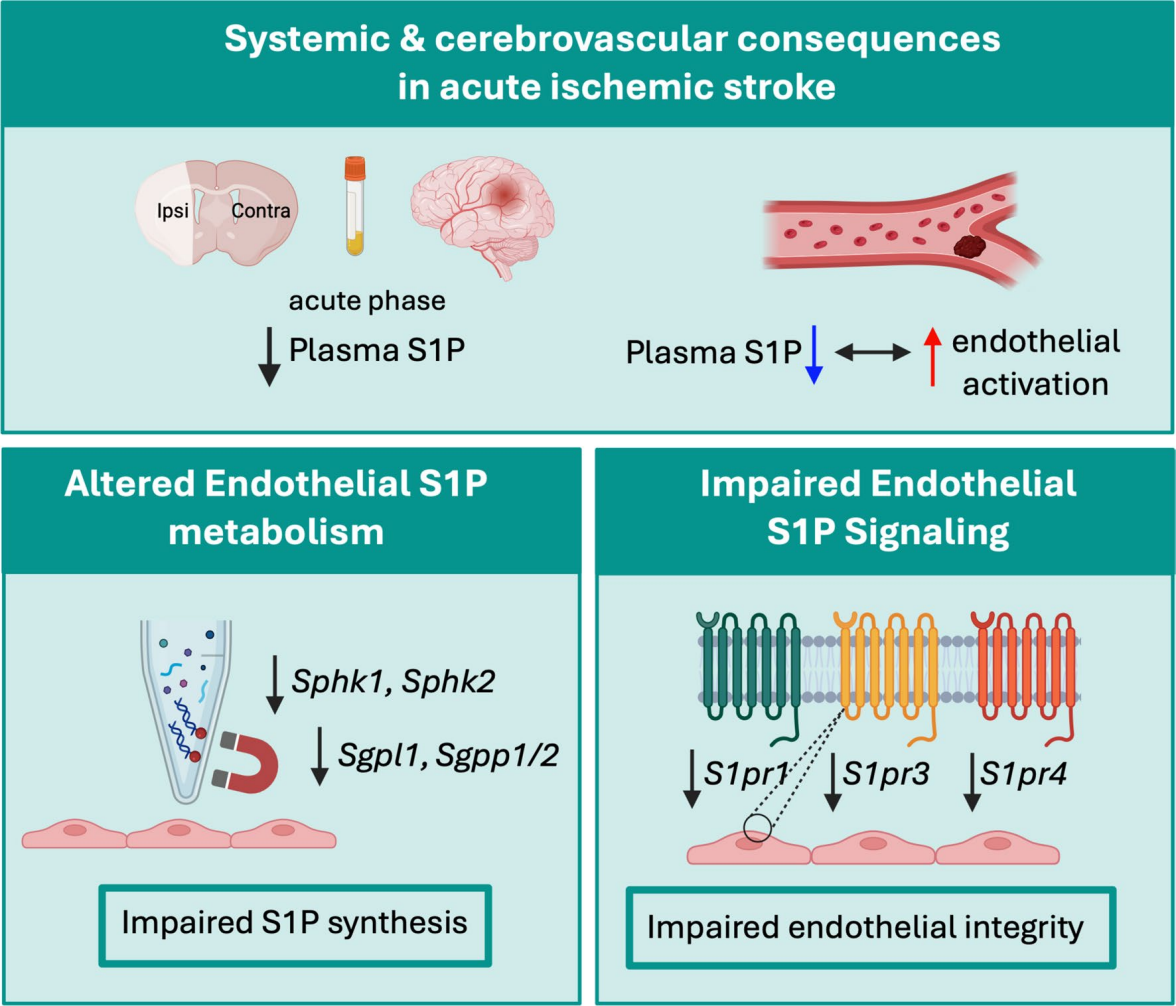

**Supplementary information:**

The online version contains supplementary material available at 10.1186/s12987-026-00828-z.

## Background

Ischemic stroke is a leading cause of long-term disability worldwide, accounting for over 116 million disability-adjusted life years [1]. Despite its prevalence, therapeutic options remain limited. The only approved acute pharmacological treatment has a narrow therapeutic window, highlighting the urgent need for new, effective stroke therapies [2]. Progress in drug development has been hindered by critical knowledge gaps in stroke pathophysiology.

Recently, the bioactive phospholipid sphingosine-1-phosphate (S1P) has gained increasing attention in cardiovascular disease, including stroke due to its involvement in vascular function responses and inflammation [3–8]. Five S1P receptors (S1PR-5) orchestrate several cellular events also relevant to stroke pathophysiology, including immune cell chemotaxis, glial cell polarization, cytokine production, vascular responsiveness, and barrier function [6, 9–13]. Specific S1PR modulation has shown beneficial effects on cerebrovascular function, cerebral blood flow and infarct size in rodent stroke models [14–16]. Particularly, endothelial S1P-S1PR1 signaling is essential for preserving the integrity of the blood-brain barrier (BBB) [17, 18]. Endothelial dysfunction and BBB breakdown are hallmarks of ischemic stroke and contribute to secondary damage through vasogenic edema, increased permeability, and neuroinflammation [19]. Alterations in S1P-S1PR1 signaling exacerbate these pathological processes, further aggravating stroke-induced damage [20]. Previous studies have reported reduced plasma S1P levels acutely following myocardial infarction [21] as well as ischemic stroke [22, 23], which is supportive of a potential link between S1P depletion and ischemic vascular injury. Reduced plasma S1P may therefore represent a biomarker candidate of endothelial injury or vascular stress [8]. Circulating S1P originates from multiple compartments, including vascular endothelium, erythrocytes, platelets, and carrier-associated plasma pools such as HDL- and albumin-bound S1P [24, 25]. Whether the reduction in plasma S1P after ischemic stroke is linked to altered brain-endothelial S1P signaling, to changes in non-endothelial S1P sources, or to a combination of these mechanisms remains unclear. In this study, we investigated whether acute ischemic stroke is associated with systemic S1P depletion alongside coordinated changes in brain-endothelial S1P pathway expression, and endothelial activation-related markers.

## Methods

### Human cohort

All human investigations conformed to the principles outlined in the Declaration of Helsinki. Patient plasma samples from subjects included in this study were selected from the Lund Stroke Recovery Study [26], which is a sub-cohort of the Lund Stroke Register that includes first-ever stroke events occurring in residents of eight municipalities in southern Sweden. The study was approved by the Regional Ethical Review Board in Lund, Sweden (Registration No. 2016/179) and the Swedish Ethics Review Board (Registration No. 2020–07047). Participants (51% female; median age 72 years) gave informed consent prior to enrollment. Based on a priori power calculations, 50 patients from the Lund Stroke Recovery Study who experienced a first ever ischemic stroke during 2021 were randomly selected and age- and sex-matched with control subjects collected during 2018–2019 as a part of the Lund Stroke Register study [27]. Plasma samples from stroke patients (*N* = 50, with 52% female) collected acutely after the stroke event (i.e., within 11-days; median 56-hours) and a 90-days follow-up, and from control subjects (*N* = 47 with 51% female, baseline characteristics of cohort see Table 1) were obtained from the regional Biobank (Biobank Sverige, Södra Sjukvårdsregionen).

**Table 1:** Baseline characteristics human ischemic stroke cohort

	Control subjects *N* = 47	Stroke patient *N* = 50	P
Females, n (%)	24 (51 %)	26 (52 %)	ns^b^
Age (median ± IQR)	72.3 (66.7–79.6)	72.9 (64.4–78.7)	ns^a^
Hypertension, n (%)	23 (49 %)	33 (66 %)	ns^b^
Diabetes mellitus, n (%) (#)	4 (8 %)	15 (30 %)	0.008^b^
Hypercholesterolemia, n (%)	39 (85 %) *	28 (56 %)	0.002 ^b^
Current smoking, n (%)	1 (2 %)	10 (20 %)	0.006 ^b^
Ischemic heart disease, n (%)	6 (13 %)	9 (18 %)	ns^b^
BMI (median ± IQR)	25.3 (23.2 - 28.1)	25.6 (23.3–28.8) **	ns^a^
CRP (mg/L) (median ± IQR)	1.50 (0.63 - 3.38) ***	1.85 (0.89–5.78) ***	ns^a^
NIHSS baseline (median ± IQR)	NA	2.5 (1.0–4.2)	-
mRS day 90 (median ± IQR)	NA	1 (0–1)	-
NIHSS day 90 (median ± IQR)	NA	0 (0–1) **	-
Plasma S1P (nmol/L)	693.3 (429.3–905.6)	587.4 (380.7–720.8)	0.003^a^

a) Mann Whitney

b) Chi-square

*) one control subject missing data

**) one stroke patient missing data

***) 3 control subjects and 6 patients missing data

(#) Definition diabetes: Fasting venous P-Glc ≥ 7 mmol/L, or fasting capillary P-Glc ≥ 7 mmol/L, or HbA1c ≥ 48 mmol/mol, or previously known diet/oral/insulin treatment for diabetes

### Clinical and laboratory assessments of human study participants

Participants’ height (cm) and weight (kg) were measured, and body mass index (BMI) was calculated. Resting blood pressure (BP; mmHg) was measured. Current smoking (yes/no), and anti-hypertensive treatment (AHT) were self-reported and/or collected from medical files. Hypertension was defined as BP ≥ 140/90 or AHT. Diabetes mellitus was defined as either self-reported treatment for diabetes mellitus (diet or use of anti-diabetic medication), HbA1c ≥ 48 mmol/mol or fasting plasma or capillary glucose ≥ 7.0 mmol/L. Hypercholesterolemia was defined as total cholesterol > 5 mmol/L, low-density lipoprotein (LDL) cholesterol > 3 mmol/L or use of lipid-lowering medication (any of statin, bezafibrate, cholestyramine, ezetimibe treatment). Each patient’s neurological status was assessed with the National Institutes of Health Stroke Scale (NIHSS) [28]. Functional status at 3 months was assessed with the modified Rankin Score (mRS) on a scale between 0 (no residual symptoms) to 6 (death) [29]. Blood samples were drawn and analyzed for plasma glucose, C-reactive protein (CRP), total serum cholesterol, high-density lipoprotein (HDL), and LDL, using standard clinical methods at the Department of Clinical Chemistry, Skåne University Hospital Lund/Malmö, which is part of a national standardization and quality control system.

### Animals

All animal experiments were approved by LANUV of North Rhine-Westphalia (AZ #81-02.04.2019.A214) and by the institutional ethics committee at Lund University (Dnr.5.8.18–08160/2021) and were conducted in accordance with ARRIVE guidelines and European animal protection laws (Directive 2010/63/EU). Adult male C57BL/6N mice between 3 - 5 months of age (22–28 g of body weight) were purchased from Charles River (Sulzfeld, Germany) or Taconic (Ejby, Denmark) and housed under 12/12 h light-dark cycle with access to food and water *ad libitum* and kept under specific pathogen-free conditions. RiboTag Rpl22tm1.1Psam mice [30] were crossbred with endothelial-specific (Cdh5) Cre recombinase mice to generate Cdh5Cre-ER(T)/RiboTag mice [31]. Following tamoxifen-induced recombination at 10 weeks of age (100 mg/kg Tamoxifen (Sigma-Aldrich #T5648) dissolved in 100 % ethanol and sunflower oil to the final concentration of 20 mg/mL was injected intraperitoneally for five consecutive days), HA-tagged Rpl22 was specifically expressed in endothelial cells. To investigate the mechanisms of endothelial cell-specific responses to ischemic stroke, mice were subjected to transient middle cerebral artery occlusion (tMCAo) three weeks after the last tamoxifen injection.

### Experimental stroke

Transient middle cerebral artery occlusion (tMCAo) was performed as previously described [32]. C57Bl6 mice were anesthetized using 3 % Isoflurane (IsoFlo vet) with a mixture of 30 % O_2_ and 70 % N_2_O. Body temperature was maintained at 37 °C ± 0.5 °C using a closed-loop rectal probe and an electric blanket (CODA monitor; Kent Scientific). The mouse was placed in the prone position, the skin on the head was disinfected with Octenisept (Schülke & Mayr) and locally anesthetized with 1 % Xylocain (Dentsply Sirona). A 1 cm long skin incision was made from the superior nuchal line to the nasion to expose the skull. A plastic fiber laser Doppler probe was attached perpendicular to the skull in a small hole (2 mm) drilled just above the left middle cerebral artery (MCA). The mouse was then placed in the supine position and a 1 mm transverse neck incision was made. The large pair of salivary glands were separated and placed to the side. The common carotid artery (CCA), internal carotid artery (ICA) and external carotid artery (ECA) were identified and dissected from the surrounding connective and fatty tissue. The CCA was carefully dissected from the vagus nerve and temporarily occluded with vessel suture (7/0; Suprama). The distal part of the ECA was permanently occluded and another loose suture was prepared close to the bifurcation. The ICA was occluded with a vascular clip. The silicone-coated monofilament (9–10 mm coating length, 0.19 ± 0.01 mm tip diameter; Doccol) was inserted into the small incision in the ECA and secured by tightening the vascular suture. The ECA was cut between the two sutures, the vascular clip was removed from the ICA, and the monofilament was advanced through the ICA to the MCA. MCA occlusion was confirmed by the drop in regional blood flow (reduction of  > 75 % from baseline) monitored by laser Doppler flowmetry (Moor Instruments, Axminster, Devon, United Kingdom). Reperfusion was induced by removing the monofilament after 60 minutes. The ECA was closed permanently and the CCA suture was removed. Reperfusion had to reach > 75 % of baseline to include the mouse in the study. The neck and head incision were closed with a silk suture (Braun). Sham surgery was performed identically except from inserting the filament and occluding the MCA. 100 µL saline was injected intraperitoneally to prevent dehydration. The mice were placed in a recovery chamber set at 37 °C. Post-surgery analgesia using Buprenorphine (0.05 mg/kg; Reckitt Benckiser Healthcare) was administered every 12 h for up to 2-days.

Permanent middle cerebral artery occlusion (pMCAo) was performed as previously described [32]. C57Bl6 mice were anesthetized using 2.5–3 % Isoflurane (Attane vet 1000 mg/g, Piramal Critical Care, Netherlands) in room air and reduced to 1.5–2 % for the surgery. The body temperature was kept at 37 °C ± 0.5 °C using a heating pad. The mouse was placed on the side and 1 cm incision was made between the left orbit and the external auditory meatus. Using electrocoagulation forceps set at 12 W, the temporal muscle was detached from the skull. 1–2 mm area of the skull above the MCA was thinned with the dental drill until the part of the skull was possible to remove. The MCA was permanently coagulated with electrocoagulation forceps set at 7 W proximal to the bifurcation followed by transection of the artery to ensure the occlusion. The temporal muscle was placed back to its original position and the incision was sutured. Sham surgery followed the same protocol except from the MCA coagulation. Mice were placed under the infrared lamp to recover from the anesthesia. Pre- (30 min before surgery) and post-surgery analgesia using Temgesic® (0.05 mg/kg; Indivior) was administered every 12 h for up to 2-days.

Infarct size was determined using 2,3,5-Triphenyltetrazolium chloride (TTC, Sigma-Aldrich Cat# 93140). Perfused brains were cut into 1 mm coronal slices using a brain matrix and incubated for 15-30 min at 37 °C in 2 % TTC dissolved in 0.9 % saline. Brain sections were imaged with a microscope, and the infarct lesion size was analyzed using ImageJ (Version: 1.54, NIH, Bethesda, USA) and presented as percentage of the ipsilateral hemisphere.

Structural characterization of the experimental stroke models, including infarct size at 1- and 3-days after tMCAo and pMCAo, is shown in Suppl. Fig. 1.

### Experimental stroke study design

C57BL/6N and Cdh5Cre-ER(T)/RiboTag mice were subjected to permanent or transient MCAo surgery. Sham surgeries were performed to account for surgery-related effects. At 1- or 3-days post-surgery, mice were anaesthetized using 2.5–3 % Isoflurane in room air and blood was collected from the *vena cava inferior* using EDTA coated tubes to prevent coagulation, and plasma was separated by centrifugation at 1,000 g for 10 min at RT. Subsequently, mice were euthanized by transcardiac perfusion with saline. The brain was extracted, separated into contralateral (control) and ipsilateral (lesioned) hemispheres and used for molecular studies; specifically, endothelial cell-specific mRNA was immunoprecipitated from ipsi- and contralateral hemispheres of Cdh5Cre-ER(T)/RiboTag mice, while hemispheres from C57BL/6N mice were subjected to vessel extraction or homogenization for Western blotting.

### Cell culture

Immortalized human brain microvascular endothelial cells (HBMECs; Innoprot Cat# P10361-IM, RRID:CVCL_YJ35) were cultured in Endothelial Cell Medium (Innoprot, Cat. # P60103) supplemented with 10 % fetal bovine serum (FBS), 1 % penicillin-streptomycin, and endothelial cell growth supplement according to the manufacturer’s instructions. Immortalized murine brain endothelial cells (b.End3; Sigma Aldrich Cat# 96091929-1VL, RRID:CVCL_0170) were cultured in Dulbecco’s Modified Eagle Medium containing 1 g/L D-glucose, sodium-pyruvate GlutaMAX™ (DMEM, Gibco Cat. #21885-025) phenol red and additional 10 % FBS) and 1 % penicillin-streptomycin. Cultures were maintained at 37 °C in a humidified incubator (Eppendorf) with 5 % CO₂ under normoxic conditions.

For experiments, HBMECs were seeded at a density of 1 × 10^5^ cells/cm^2^ in culture plates coated with fibronectin (Innoprot Cat # P8248) and allowed to reach 70 % confluence in growth media before media was exchanged to DMEM and cells were allowed to equilibrate for a minimum of 48 h. At ~ 90 % confluency, HBMECS and b.End3 cells were used for experimentation, for which media was changed to DMEM (Cat#A14430-01) with 4 mM L-glutamine, but without glucose, phenol red and sodium-pyruvate. To induce hypoxic stress, cells were transferred to a humidified hypoxia incubator (PHCbi O_2_/CO_2_ incubator MCO-50 M) equilibrated to 1 % O₂, 5 % CO₂, and 94 % N₂ for 24 h at 37 °C. Normoxic controls were maintained in parallel in standard incubators (Eppendorf) at 21 % O₂ and 5% CO₂. Following hypoxia exposure, cells were immediately harvested for RNA extraction. Each experiment was performed in triplicates and repeated 3 times using different cell passages.

### S1P quantitation

S1P was extracted by mixing 10 µL of plasma with 90 µL of ice-cold methanol containing 22.2 nM S1P-D7 (Avanti Polar Lipids/Merck, Darmstadt, Germany) as internal standard. After incubation on ice for 30 min precipitate was removed by centrifugation (20 000 g for 10 min at 4 °C). The supernatant was analyzed by LC-MS/MS on a 6495 QQQ instrument (Agilent Technologies, Kista, Sweden) as previously described [7, 8, 33, 34]. Extracts were separated on a 2.1 × 50 mm Acquity UPLC Peptide HSS T3 C18 column (Waters, Sollentuna, Sweden) at a flow rate of 0.5 mL/min using eluents (A) water/0.1 % formic acid/1 mM ammonium formate, and (B) methanol/0.1 % formic acid/1 mM ammonium formate, with a gradient of 20 % A and 80 % B to 100 % B over 2 min, followed by 100 % B for 6 min. By multiple reaction monitoring, MS/MS transitions of *m/z* 380 to 264 (with 380 to 82 as qualifier) for S1P, and 387 to 271 (with 387 to 82 as qualifier) for S1P-D7, were measured. A calibration curve consisting of 7 concentrations in the range of 0.1 to 2.4 µM S1P in 4 % BSA was generated in triplicates and measured during each session. Additionally, 3 plasma samples were used as quality control samples and measured during each session to ensure reproducibility.

### Immunoprecipitation of mRNA with RiboTag

Each brain hemisphere from Cdh5Cre-ER(T)/RiboTag was homogenized in 1 mL polysome buffer (PSB; 50 mM Tris pH 7.5, 100 mM KCl, 12 mM MgCl2, 1 % Nonidet *P*-40, 1 mM Dithiothreitol, 3.75 mL/mL RNase inhibitor, 100 mg/mL Cycloheximide, 2x Protease inhibitor, 1x Phosphatase inhibitor) using Precellys. Supernatant 1 (S1) was prepared by centrifugation at 10,000 g for 10 min at 4 °C. Total mRNA was used as control and prepared by mixing 100 mL of S1 with 700 mL QIAzol (Qiagen, 79306) to subsequently extract mRNA. The rest of S1 was pre-cleared by incubation with Protein G Dynabeads (PGDB; Life Technologies, 10004D) for 30 min at 4 °C. Pre-cleared S1 was transferred to a tube containing anti-HA antibody (Sigma-Aldrich Cat# 11583816001, RRID:AB_514505) and incubated for 45 min at 4 °C. The lysate with the antibody was then added to a new volume of PGDB and incubated for 80 min at 4 °C. In the last step, samples were placed on the magnetic rack to allow PGDB to adhere to the wall and the unbound fraction was discarded. PGDB were washed three times with high salt buffer (HSB; 50 mM Tris pH 7.5, 300 mM KCl, 12 mM MgCl2, 1 % Nonidet *P*-40, 1 mM Dithiothreitol, 1.25 mL/mL RNase inhibitor, 10 mg/mL Cycloheximide, 0.5x Protease inhibitor, 1x Phosphatase inhibitor) followed by additional three washes with extra high salt buffer (EHSB; 50 mM Tris pH 7.5, 300 mM KCl, 300 mM NaCl, 12 mM MgCl2, 1 % Nonidet *P*-40, 1 mM Dithiothreitol, 1.25 mL/mL RNase inhibitor, 10 mg/mL Cycloheximide, 2x Protease inhibitor, 1x Phosphatase inhibitor) to reduce the background. Actively translated mRNA was extracted with QIAzol and RNeasy Micro Kit (Qiagen, 74,004) according to the manufacturer’s instructions. In the last step, mRNA was eluted with 28 mL of RNase-free water. RNA quality was evaluated using Agilent RNA 6000 pico Kit (Agilent Technologies, 5067–1513) on an Agilent 2100 Bioanalyzer Instrument (RRID:SCR_018043) or Roche LightCycler 480 Real Time PCR System (RRID:SCR_018626). To avoid degradation, mRNA was directly transcribed to cDNA and stored at −20 °C.

### RNA isolation from cell culture

RNA was isolated using TriZol Reagent (Thermo Fisher Scientific, Cat#15596018) according to manufacturer’s instructions. One µg of mRNA was reverse transcribed into cDNA using the High-Capacity cDNA Reverse Transcription Kit (Thermo Fisher, Cat#4368814) in an T100 Thermal Cycler (Bio-Rad, Hercules, CA, USA). The resulting cDNA was diluted 1:12.5 and used as template for quantitative RT-PCR.

### Quantitative RT-PCR

Gene expression (see Suppl. Table 1 for primer pairs used in qPCR) was detected using SYBR Green PCR Master Mix (Thermo Fisher Scientific, #4369702) and Roche LightCycler 480 Real Time PCR System (RRID:SCR_018626). All samples were run in triplicates and the relative gene expression was calculated from the standard curve and normalized to the housekeeping gene L14 or GPI. qPCR data are generally presented as values normalized to the relevant control condition or as relative change to normoxia and where indicated, as normalized transcript abundance relative to the housekeeping gene; the format used is specified in each figure and supplementary legend.

### Vessel parenchyma fractionation

Brain tissue fractionation was performed as described previously [35]. Briefly, each hemisphere was homogenized in 1 mL B1 (HBSS with 10 mM HEPES) with a 21 G cannula mounted on a syringe and centrifuged at 2,000 g for 10 min at 4 °C. The supernatant representing the vessel-depleted brain fraction was mixed with equal volume of 2x RIPA buffer (20 mM Tris pH 8.0, 2 mM EDTA, 2 % Triton X-100, 0.2 % sodium de-oxycholate, 0.2 % SDS, 280 mM NaCl) supplemented with 1x protease (Thermo Fisher Scientific, 87785) and 1x phosphatase inhibitors (Thermo Fisher Scientific, 78420). Protein was isolated as described below in Western blot section. Pellets containing vessel-rich fractions were resuspended with B2 (B1 with 18 % dextran 70000; Sigma Aldrich, 31390), mixed thoroughly and centrifuged at 4,400 g for 15 min at 4 °C. Pellets were then resuspended in B3 (B1 with 1 % BSA) and transferred onto a 20 µm cell strainer (Pluriselect, 43–10020) followed by centrifugation at 200 g for 1 min at 4 °C to collect the vessel-rich fraction. The vessel-rich fraction captured on the strainer was washed twice with B3 and subsequent centrifugation. The vessel-rich fraction was collected by resuspending in B3 and pelleted by centrifugation at 2,000 g for 5 min at 4 °C. Finally, the vessel-rich fraction was washed with B1 to remove BSA.

### Immunofluorescence

Purified vessels were fixed with 4 % PFA for 30 min and permeabilized with 0.2 % (v/v) Triton X-100 in PBS for 15 min at room temperature. After PBS washing and blocking with blocking reagent (Roche, Sweden, Cat#11096176001) for 30 min, samples were immune-stained against CD31 (R and D Systems Cat# AF3628, RRID:AB_2161028, 1:200), GFAP and SphK1 (Thermo Fisher Scientific Cat# 13–0300, RRID:AB_2532994, 1:200; abcam Cat# ab71700, RRID:AB_1270981, 1:200) or SphK2 (Proteintech Cat# 17096–1-AP, RRID:AB_10598479, 1:200) in Poly-D-Lysine-coated 4-well cell culture slides (Sarstedt, Cat# 94.6170.402) overnight at 4 °C. Samples were washed with PBS, incubated with secondary antibody (donkey anti-rabbit Alexa Fluor 568, Abcam Cat# ab175470, RRID:AB_2783823, 1:500; donkey-anti rat 488, Abcam Cat# ab150153, RRID:AB_2737355, 1:500; or donkey-anti goat 647, Abcam Cat# ab150131, RRID:AB_2732857, 1:500) at room temperature, and mounted with Fluoromount-G with DAPI (Thermo Fisher, Cat#00–4959-52). Images were acquired using a Leica STELLARIS 5 microscope (RRID:SCR_024663). Endothelial expression of SphK1 and SphK2 was assessed using a scoring system. Signal intensity was graded on a scale from 0 to 4 (0 = no detectable signal, 1 = low, 2 = moderate, 3 = strong, 4 = very strong). Endothelial cells were identified based on morphological and immunofluorescent criteria. To define nuclear morphology cell nuclei were visualized using DAPI staining, and endothelial identity was confirmed by CD31 positivity. To exclude non-endothelial cells, particularly astrocytes, regions showing GFAP co-localization were excluded from the analysis.

### Western blotting

Tissue homogenates or purified vessel-rich fractions were solubilized in RIPA buffer (50 mM Tris-HCl pH 8.0, 5 mM EDTA, 1 % Triton-X, 1 % sodium-deoxycholate, 0.1 % SDS, 150 mM NaCl) supplemented with phosphatase (Abcam, Cat# ab201112) and protease inhibitors (Roche, Cat# 04693124001) for 30 min on ice. Insoluble material was removed by centrifugation at 20,000 g at 4 °C for 10 min. Brain vessels were separated from parenchyma as previously described [35]. Briefly, one brain hemisphere was minced to small pieces and homogenized in HBSS containing 10 mM HEPES using a long 21 G cannula mounted on a 2 mL syringe. The homogenate was transferred to a 2 mL tube and centrifuged in a fixed-angle rotor at 2,000 g and 4 °C for 10 min. The supernatant, representing the parenchyma fraction, was taken off and mixed with an equal volume of 2x RIPA buffer including protease and phosphatase inhibitor cocktails. The pellet containing the vessels was resuspended in HBSS containing 10 mM HEPES and 18 % (w/v) dextran (Mr ≈ 70,000), and centrifuged at 4,400 g and 4 °C for 15 min. The supernatant including the myelin layer was carefully removed and the pellet was resuspended in HBSS containing 10 mM HEPES and 1 % (w/v) BSA. Brain vessels were collected on a 20 µm mini cell strainer (pluriSelect, Leipzig, Germany) by centrifugation at 200 g for 1 min in a swinging-bucket rotor. Vessels were washed twice with HBSS containing 10 mM HEPES and 1 % (w/v) BSA. The purified vessels were transferred to a 1.5 mL tube, and sedimented at 2,000 g and 4 °C for 5 min. To remove BSA the supernatant was discarded and the pellet was carefully resuspended in HBSS containing 10 mM HEPES, followed by centrifugation at 2,000 g and 4 °C for 5 min. Supernatant was removed completely and the vessels were mixed with 100 µl RIPA buffer containing 10 mM Tris (pH 8.0), 1 mM EDTA, 1 % Triton X-100, 0.1 % sodium deoxycholate, 0.1 % SDS, 140 mM NaCl and protease and phosphatase inhibitor cocktails (Merck, Darmstadt, Germany), and homogenized in a glass micro homogenizer (Radnoti, Dublin, Ireland) with at least 20 strokes. Protein concentration was measured using Pierce BCA Protein Assay Kit (Thermo Fisher, Cat#23227) and 30 µg of protein (5 µg for vessels) was mixed with 4x sample buffer (0.2 M Tris pH 6.8, 8 % SDS, 40 % (v/v) glycerol, 20 % (v/v) β-mercaptoethanol, 0.02 % (w/v) bromophenol blue) and heated for 10 min at 95 °C.

Proteins were loaded on 4–15 % SDS-PAGE stain free mini gels and separated at 100 V for 2 h. Proteins were transferred onto PVDF or low fluorescent PVDF membranes (Bio-Rad) using a Bio-Rad Trans-Blot Turbo Transfer System (RRID:SCR_023156). Membranes were blocked for 1 h in 5 % non-fat dry milk (in phosphate-buffered saline containing 1 % Tween-20 (PBST); 137 mM NaCl, 2.7 mM KCl, 10 mM Na_2_HPO_4_, 1.8 mM KH_2_PO_4_; pH 7.4) at RT and followed by incubation with primary antibody anti-S1PR1 (Thermo Fisher Scientific Cat# PA1-1040, RRID:AB_2184729, 1:1,000), anti-S1PR4 (Novus, Cat# NBP2-24500, RRID:AB_3086741, 1:1,000), anti-albumin (R&D Systems, Cat# AF3329, RRID:AB_3644370;1:1,000), VE-Cadherin (abcam, Cat# ab33168, RRID:AB_870662), anti-beta-actin (Sigma-Aldrich, Cat# MAB1501, RRID:AB_2223041; 1:5,000) and anti-beta-tubulin (Sigma-Aldrich, Cat# T4026, RRID:AB_477577, 1:5,000) overnight at 4 °C. Next, membranes were incubated with HRP-conjugated goat anti-mouse (Dianova, Cat# 115-035-062, 1:10,000), goat anti-rabbit (Cell Signaling Cat# 7074, RRID:AB_2099233, 1:10,000) or donkey-anti goat IgG Cross-Adsorbed Secondary Antibody, Alexa Fluor 594 (Thermo Fisher Scientific Cat# A-11058, 1:1,000, RRID:AB_2534105) antibody for 1 h at RT. Proteins were visualized by enhanced chemiluminescence (SuperSignal West femto Maximum Sensitivity Substrate Thermo Fisher, Cat# 34095) using a BioRad ChemiDoc MP Imaging System (RRID:SCR_019037). Relative VE-Cadherin, S1PR1, S1PR4 and serum-albumin protein expression normalized to total lane protein, b-actin or b-tubulin were analyzed using Image Lab Software 6.1.0 (RRID:SCR_014210). Total lane protein was determined by total lane protein on stain free membranes before antibody incubation.

### ELISA

P-selectin concentration was determined in EDTA plasma using a commercially available human P-selectin/CD62P ELISA kits (R&D, DPSE00) and mouse sP-Selectin/CD62P Quantikine ELISA kits (R&D, MPS00) as per manufacturer’s instructions.

### Statistics

Statistical analyses of human data were performed using SPSS (RRID:SCR_002865; version 28). Normality was assessed using the Shapiro-Wilk test. Comparisons between two independent groups were performed using the Mann-Whitney U test or Student’s t-test, as appropriate. Categorical variables were analyzed using the χ^2^ test. Associations between variables were assessed using Spearman rank correlation analysis with two-tailed significance testing and calculation of Spearman’s correlation coefficient. In addition, multivariable logistic regression was performed to assess the independent association between plasma S1P and acute ischemic stroke status. Stroke status (patient vs control) was used as the dependent variable, and plasma S1P was entered as the independent variable of interest together with sex, age, hypertension, diabetes, hypercholesterolemia, smoking, ischemic heart disease, and BMI as covariates. Smoking was entered as a binary categorical variable coded as current smoker = 1 and ex-/never smoker = 2; this coding was retained from the clinical dataset and was considered in interpretation of the corresponding regression coefficient and odds ratio. Regression coefficients (B), odds ratios (OR), and corresponding *p* values are reported. For interpretability, rescaled ORs were additionally calculated for selected continuous variables, specifically plasma S1P per 100-unit increase and age per 10-year increase.

Sample sizes for murine experiments were based on prior experience with these stroke models and endpoint variability [32, 35, 36]. No animals or data points were excluded unless prespecified technical or surgical criteria were not met (for example, failure to achieve a reduction in cerebral blood flow of at least 75 % from baseline in the tMCAo model). Only male mice were used; therefore, sex as a biological variable was not tested in the experimental studies. Exact *N* values are provided in the figure legends. Data from mouse experiments and in vitro studies were analyzed using GraphPad Prism (RRID:SCR_002798; version 10). The sample size (N) denotes the number of animals or independent experiments for in vitro studies. Data are presented as mean ± SEM for normally distributed variables or as median ± interquartile range for non-normally distributed data evaluated using the Shapiro-Wilk test. Comparisons between two dependent groups were performed using paired Student’s t-test or Wilcoxon signed-rank test, as appropriate based on data distribution. For comparisons involving multiple groups, two-way analysis of variance (ANOVA) with Sidak’s post hoc correction was applied, with experimental group and time post-stroke included as factors. Because multiple targets within the same experiment were analyzed, false discovery rate correction (Benjamini-Hochberg) was applied within each predefined target panel. *p* values < 0.05 were considered statistically significant.

## Results

**Fig. 1:**
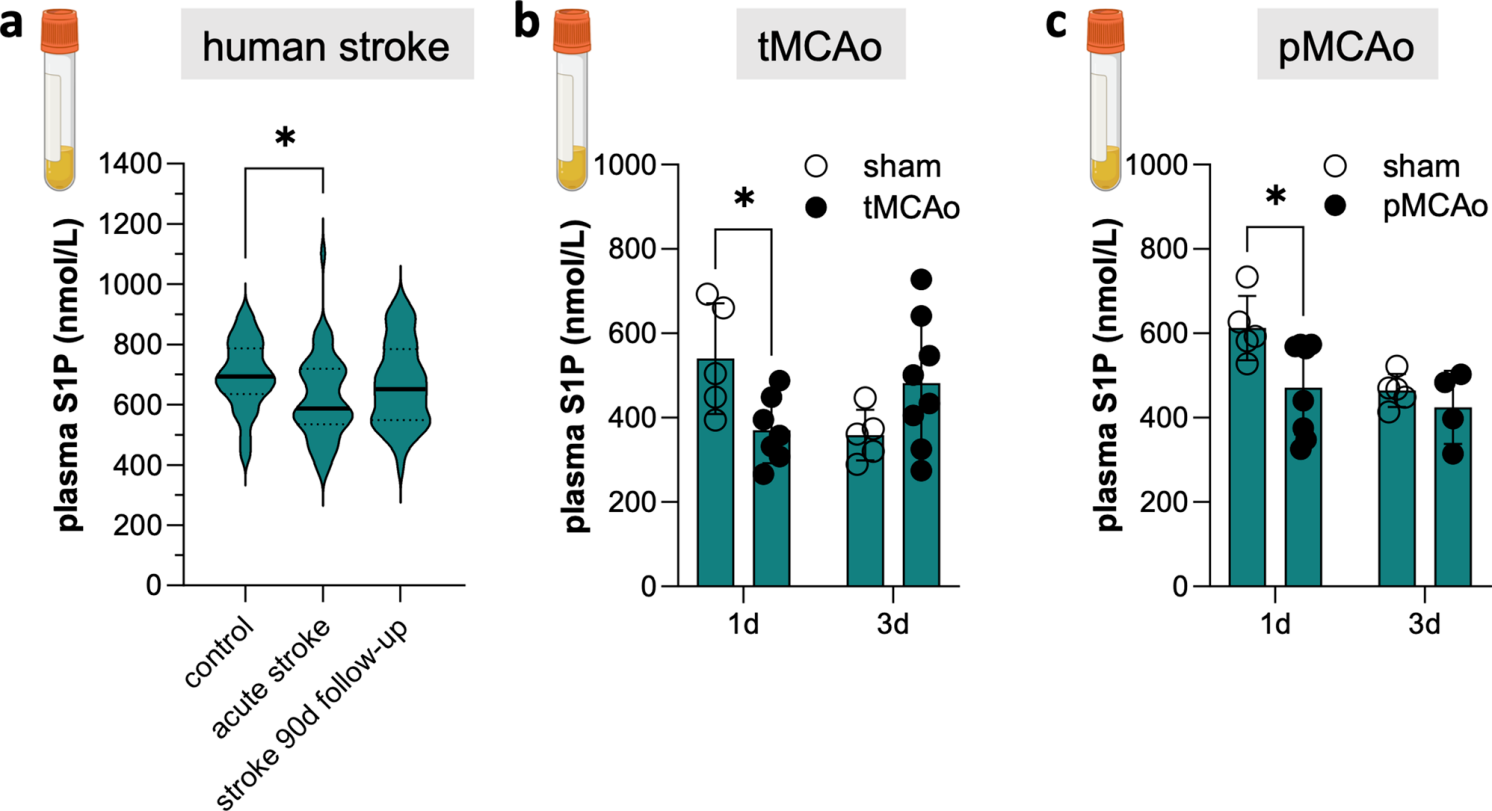
Reduced plasma S1P levels in the acute phase after ischemic stroke. (**a**) Plasma sphingosine-1-phosphate (S1P) concentrations in healthy controls, patients with acute ischemic stroke, and at 90-day follow-up. *N* = 47 for controls and *N* = 50 for ischemic stroke samples. (**b**) Plasma S1P levels at 1- and 3-days after transient middle cerebral artery occlusion (tMCAo) in mice. *N* = 5 for sham controls; *N* = 7 for tMCAo. (**c**) Plasma S1P levels at 1- and 3-days after permanent middle cerebral artery occlusion (pMCAo) in mice. *N* = 5 for sham controls; *N* = 8 for pMCAo. Data are presented as median ± interquartile range (IQR) and analyzed using the Mann–Whitney test (**a**) or as mean ± SEM and analyzed by two-way ANOVA (**b, c**). * *p* < 0.05. *S1P – sphingosine-1-phosphate, pMCAo – permanent middle cerebral artery occlusion, tMCAo – transient middle cerebral artery occlusion*

Plasma S1P levels were significantly lower in patients with acute ischemic stroke (*N* = 50) compared to age- and sex-matched controls (*N* = 47; 587.4 nmol/L vs. 693.3 nmol/L, *p* = 0.003 Table 1; Fig. 1a). At 90-days post-stroke, plasma S1P levels were neither significantly different compared to the acute phase (651.9 nmol/L vs. 587.4 nmol/L, *p* = 0.104; Fig. 1a) nor to control levels (651.9 nmol/L vs. 693.3 nmol/L, *p* = 0.431; Fig. 1a). In multivariable logistic regression adjusting for sex, age, hypertension, diabetes, hypercholesterolemia, smoking, ischemic heart disease, and body mass index, lower plasma S1P remained independently associated with acute ischemic stroke status (B = −0.005, OR = 0.995 per unit increase, *p* = 0.032; Table 2a), corresponding to an OR of 0.606 per 100-unit increase in plasma S1P. Spearman correlation analysis revealed no significant association between acute plasma S1P levels and clinical stroke severity, as measured by the National Institutes of Health Stroke Scale (NIHSS) at stroke onset (Table 2b). Furthermore, neither acute nor follow-up S1P levels correlated with functional outcome, assessed by the modified Rankin Scale (mRS) or NIHSS at 90-day follow-up (Table 2b). Additionally, S1P levels showed no correlation with C-reactive protein (CRP) levels, a surrogate marker of systemic inflammation in stroke patients during the acute phase (Table 2b).

**Table 2:** Human cohort analyses - associations between plasma S1P and clinical and laboratory parameters

a	B	p-value	OR	**OR**_**rescaled**_
acute plasma S1P (nmol/mL)	−0.005	0.032	0.995	OR_100_ = 0.606
sex	0.938	0.118	2.554	-
age	−0.036	0.209	0.964	OR_10_ = 0.693
hypertension	0.667	0.253	1.969	-
diabetes	1.083	0.154	2.955	-
hypercholesterolemia	−1.496	0.017	0.224	-
Not smoking (ex or never)	−3.200	0.008	0.041	-
Ischemic heart disease	0.786	0.361	2.155	-
BMI	−0.022	0.729	0.979	-

**b**	**Spearman ρ** **N = 50**	**p-value**		
Acute plasma S1P (nmol/mL)				
Acute NIHSS	0.073	0.616		
Follow-up mRS	0.185	0.199		
CRP (mg/L)	0.081	0.600		
Follow-up NIHSS	0.012	0.932		
Follow-up plasma S1P (nmol/mL)				
Follow-up NIHSS	−0.097	0.507		
Follow-up mRS	−0.071	0.632		

**(a)** Multivariable logistic regression of factors associated with acute ischemic stroke status in the acute case-control cohort. Stroke status (case vs control) was used as the dependent variable, with plasma S1P as the independent variable of interest and sex, age, hypertension, diabetes, smoking, ischemic heart disease, and BMI as covariates. For continuous variables, rescaled ORs are additionally shown for interpretability (plasma S1P per 100-unit increase; age per 10-year increase). Smoking was coded as current smoker = 1 and ex-/never smoker = 2; therefore, the corresponding OR reflects the change from current smoking to ex-/never smoking. Only participant with complete records for all variables were included (*N* = 46 for control, *N* = 49 for stroke patients). **(b)** Spearman correlation analysis in stroke patients sampled during the acute phase and at 90-day follow-up (*N* = 50, with 52% female). *CRP = C-reactive protein; mRS = modified Rankin Scale; NIHSS = National Institutes of Health Stroke Scale; OR = odds ratio; S1P = sphingosine-1-phosphate*

To further explore the role of circulating S1P in ischemic stroke, we employed experimental stroke models using middle cerebral artery occlusion (MCAo) in mice. Plasma S1P levels were significantly lower in MCAo mice at 24 h following both permanent and transient MCAo compared with their respective sham-operated controls (Fig. 1b–c; Suppl. Table 2), mirroring the findings observed in stroke patients. However, comparison with naïve reference values revealed model-specific differences. In the tMCAo model, plasma S1P at 24 h was reduced below both sham and naïve levels, whereas in the pMCAo model, plasma S1P was lower than in sham-operated mice but remained within the naïve range (Suppl. Table 2). At later time points, plasma S1P levels did not significantly differ from those of the respective controls or naïve mice (Fig. 1b–c; Suppl. Table 2).

**Fig. 2:**
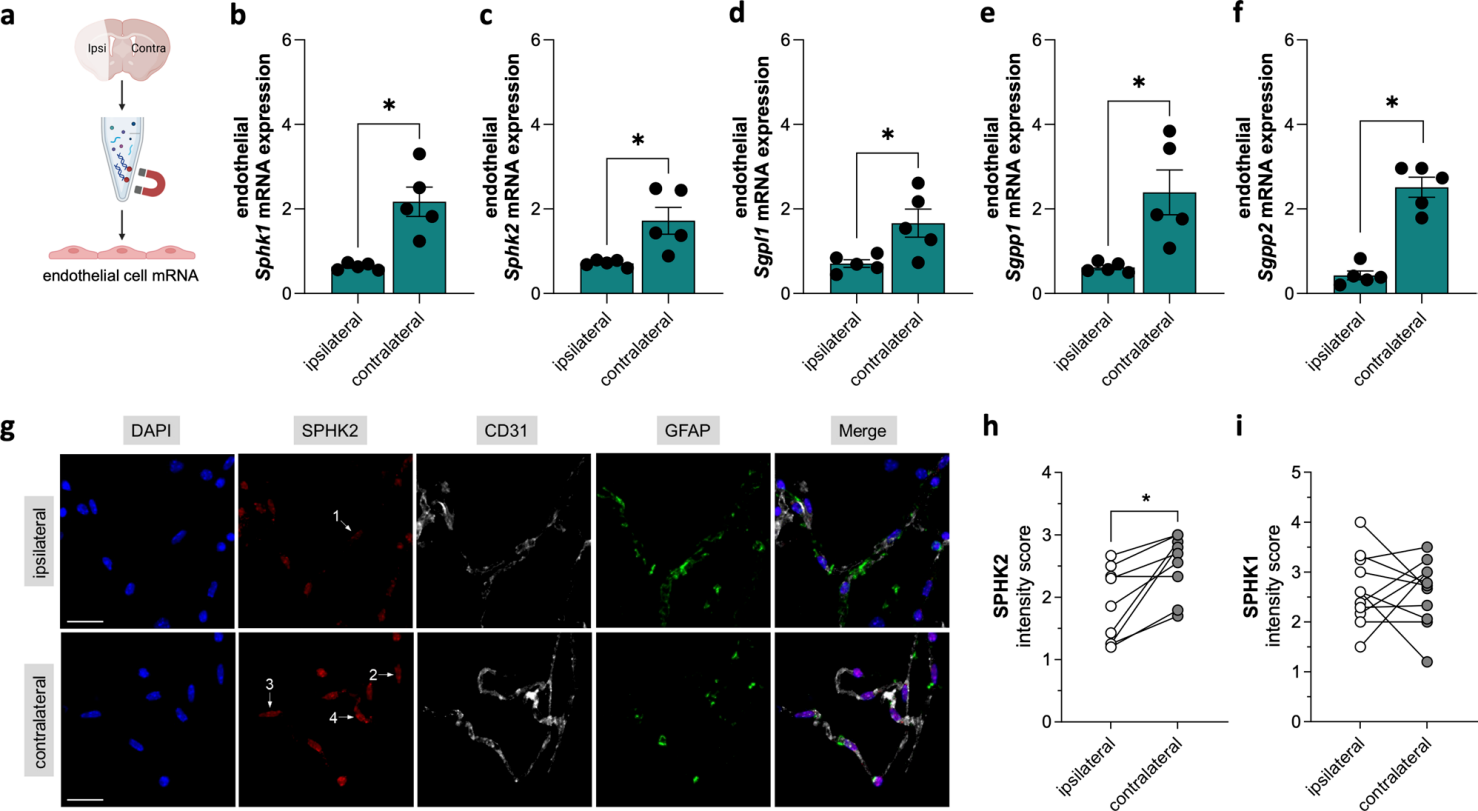
Stroke-induced regulation of S1P metabolic enzymes in the cerebral endothelium. (**a**) Schematic illustration of the endothelial-specific RiboTag strategy using Cdh5^Cre-ER(**T**) mice to isolate endothelial mRNA from brain tissue. Endothelial mRNA expression of *Sphk1* (**b**), *Sphk2* (**c**), *Sgpl1* (**d**), *Sgpp1* (**e**), and *Sgpp2* (**f**) in ipsilateral and contralateral hemispheres 1-day after transient middle cerebral artery occlusion (tMcao). Endothelial-specific transcripts were obtained by RiboTag immunoprecipitation. (**g**) Representative immunofluorescence images of cerebral vessels isolated 1-day after permanent middle cerebral artery occlusion (pMCAo) stained for 4′,6-diamidino-2-phenylindole (DAPI), cluster of differentiation 31 (CD31), glial fibrillary acidic protein (GFAP) and sphingosine kinase 2 (SPHK2). (**h-i**) Quantification of endothelial (**h**) SPHK2 and (**i**) SPHK1 immunofluorescence intensity (score: 0 = no detectable signal, 1 = low, 2 = moderate, 3 = strong, 4 = very strong, arrows in SPHK2 staining are indicating scoring examples) in ipsilateral compared to contralateral vessels. *N* = 5 for all readouts. Data are presented as mean ± SEM. * *p* < 0.05 after paired t-tests. *CD31 – cluster of differentiation 31, GFAP – glial fibrillary acidic protein, S1P – sphingosine-1-phosphate, Sphk – sphingosine kinase, Sgpl – sphingosine lyase, Sgpp – sphingosine phosphatase*

To assess whether these changes in circulating S1P were accompanied by alterations in the endothelial S1P pathway, we next analyzed the brain endothelial translatome using endothelial-specific RiboTag mice (Cdh5^Cre-ER (T)/RiboTag; Fig. 2a) subjected to tMCAo. We observed a significant reduction in endothelial *Sphk1* transcript abundance in the ipsilateral compared with the contralateral hemisphere at 1-day post-stroke (Fig. 2b), whereas no hemispheric difference was detected at 3-days post-stroke (Suppl. Fig. 2b). Endothelial *Sphk2* transcript abundance was likewise reduced in the ipsilateral hemisphere at 1-day post-stroke (Fig. 2c), indicating an acute suppression of endothelial S1P synthesis-related gene expression following ischemic injury. Transcript enrichment analyses showed that *Sphk1* and *Sphk2* were enriched in endothelial isolates relative to total tissue (4.2-fold and 1.9-fold, respectively; Suppl. Fig. 3). While *Sphk2* total-tissue expression did not change after stroke, *Sphk1* transcript abundance was increased in ipsilateral total-tissue extracts (Suppl. Table 3), indicating isoform-dependent differential regulation in endothelial-enriched versus total brain fractions. To complement the transcript-level analyses, cerebral vessels were isolated 1-day after pMCAo and immunostained for cluster of differentiation 31 (CD31) and glial fibrillary acidic protein (GFAP) together with SPHK1 or SPHK2. In these preparations, endothelial SPHK2 intensity (mainly associated with nuclear structures) was 26 % lower in vessels isolated from ipsilateral compared with contralateral vessels (Fig. 2g–h), providing protein-level support for acute suppression of endothelial S1P generating proteins after ischemic stroke. In contrast, endothelial SPHK1 intensity was less prominent and more associated to GFAP signals (Suppl. Fig. 4). Analysis of endothelial-associated SPHK1 did not yield significant differences between ipsilateral and contralateral vessels (Fig. 2i, Suppl. Fig. 4), indicating a selective downregulation of endothelial SPHK2 at the protein level.

All three major S1P-degrading enzymes, *Sgpl1, Sgpp1, and Sgpp2*, were enriched in endothelial isolates relative to total tissue (> 2-fold; Suppl. Fig. 3). mRNA expression of these enzymes was significantly reduced in the ipsilateral endothelium at 1-day post-stroke (Fig. 2d–f), whereas total-tissue extracts showed isoform-specific responses, with ipsilateral elevation of *Sgpl1* and reduction of *Sgpp1* (Suppl. Table 3). By 3-days post-stroke, endothelial expression levels of these S1P-degrading enzymes no longer differed between hemispheres (Suppl. Fig. 2c-d). Together, these findings show that endothelial transcripts related to both S1P synthesis and degradation are acutely reduced after stroke.

**Fig. 3:**
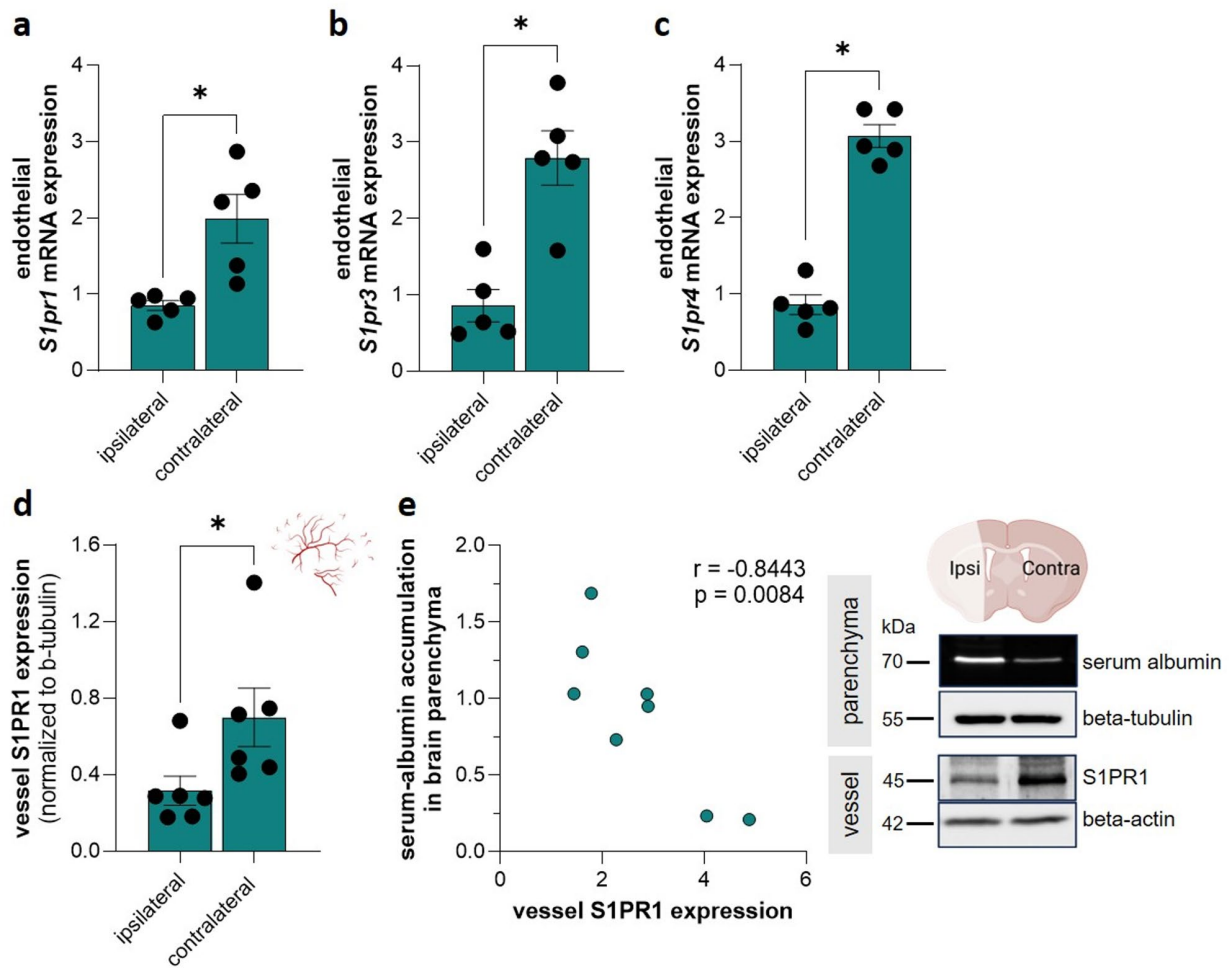
Endothelial cell-specific expression of S1P receptors after transient middle cerebral artery occlusion. Endothelial mRNA expression of *S1pr1* **(a)**, *S1pr3* **(b)**, and *S1pr4* **(c)** was measured in RiboTag isolates from the ipsilateral and contralateral hemispheres 1-day after transient middle cerebral artery occlusion (tMCAo). *N* = 5 for all readouts. **(d)** Protein levels of S1PR1 in microvessel-enriched fractions from ipsilateral and contralateral hemispheres after permanent middle cerebral artery occlusion (pMCAo). **(e)** Correlation between vessel S1PR1 expression and parenchymal serum albumin accumulation in the pMCAo model, as a marker of blood-brain barrier leakage. Representative western blot images are shown for each protein. *N* = 6 for all readouts. Data are presented as mean ± SEM. * p < 0.05 after paired t-tests.* S1P* –-, *sphingosine-1-phosphate,* *contra – contralateral, ipsi – ipsilateral, S1P – sphingosine-1-phosphate,* *S1pr – S1P receptor*

The reduction in plasma S1P levels acutely after stroke was accompanied by downregulation of S1P receptor mRNA expression in the ischemic cerebral endothelium. Specifically, endothelial *S1pr1*, *S1pr3* and *S1pr4* transcripts were lowered in ipsilateral compared to contralateral hemisphere at 1-day post-stroke (Fig. 3a–c). *S1pr2* and *S1pr5* mRNA expression was below the detection limit in both compartments or the endothelial compartment, respectively (Suppl. Table 3). Transcriptomic enrichment analyses further confirmed the endothelial predominance of specific S1P receptors: *S1pr1* and *S1pr4* mRNA levels were enriched 9.2-fold and 50-fold, respectively, in endothelial cells compared with total brain extracts (Suppl. Fig. 3f,h). Both *S1pr1* and *S1pr4* were selectively reduced in the ipsilateral cerebral endothelium after stroke, whereas their expression in total tissue remained unchanged (Suppl. Table 3). *S1pr3* transcripts were enriched in endothelial cells (4.3-fold; Suppl. Fig. 3 g) and were likewise reduced in the ipsilateral endothelium (Fig. 3b); however, *S1pr3* expression was increased in total ipsilateral tissue (Suppl. Table 3), indicating concomitant upregulation in non-endothelial cell populations during the acute post-stroke phase [32]. Taken together, these data demonstrate a stroke-associated, endothelial-specific reduction of S1P receptor subtypes 1 and 4, suggesting altered brain-endothelial S1P signaling in the ischemic hemisphere [37, 38]. This is further supported by an inverse relationship between cerebrovascular S1PR1 protein expression and serum albumin accumulation in vessel-depleted brain parenchyma (Fig. 3d–e), consistent with BBB leakage in the pMCAo model. Here, S1PR4 protein was below detection limit in vessel enriched fractions, and whole-hemisphere protein expression did not differ between the ipsi- and contralateral sides (Suppl. Fig. 5a-b). Similarly, whole tissue S1PR1 protein expression yielded no difference between the hemispheres (Suppl. Fig. 5c-d).

**Fig. 4:**
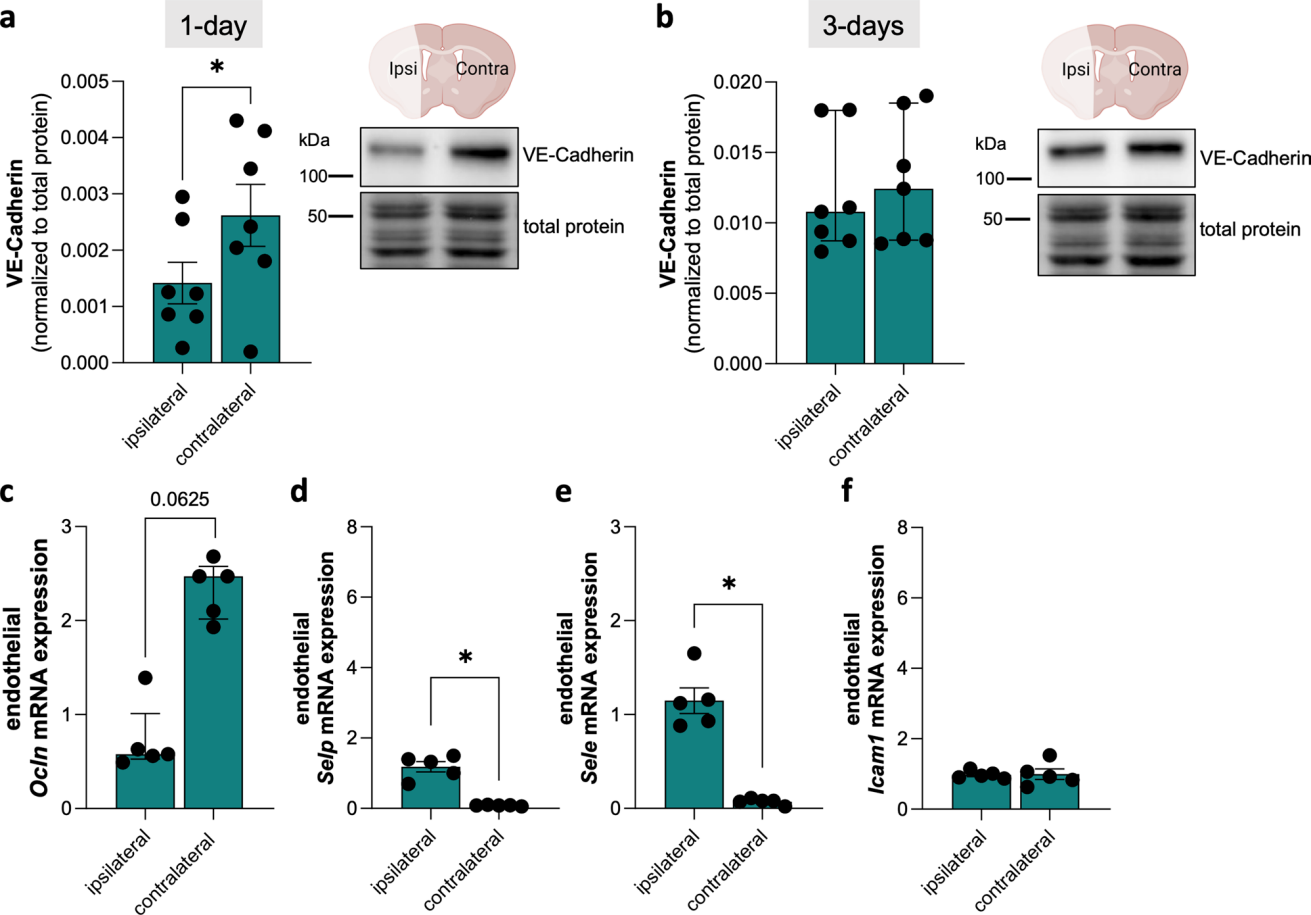
Endothelial barrier and activation markers after permanent middle cerebral artery occlusion. Protein expression of VE-cadherin in whole-hemisphere homogenates at 1-day (**a**) and 3-days (**b**) after permanent middle cerebral artery occlusion (pMCAo) is shown, including representative western blot images. Endothelial mRNA expression of occludin (*Ocln*) (**c**), P-selectin (*Selp*) (**d**), E-selectin (*Sele*) (**e**), and intercellular adhesion molecule 1 (*Icam1*) (**f**) was measured in RiboTag isolates from ipsilateral and contralateral hemispheres of transient middle cerebral artery occlusion (tMCAo). *N* = 5 for all readouts. Data are presented as mean ± SEM (a, d, e, f) or median ± interquartile range (**b, c**). * *p* < 0.05 after paired t-tests. *Contra – contralateral, intercellular adhesion molecule 1 - Icam1, ipsi – ipsilateral, ocln – occludin*, *sele – E-selectin, selp – P-selectin, VE-Cadherin – vascular endothelial-cadherin*

**Fig. 5:**
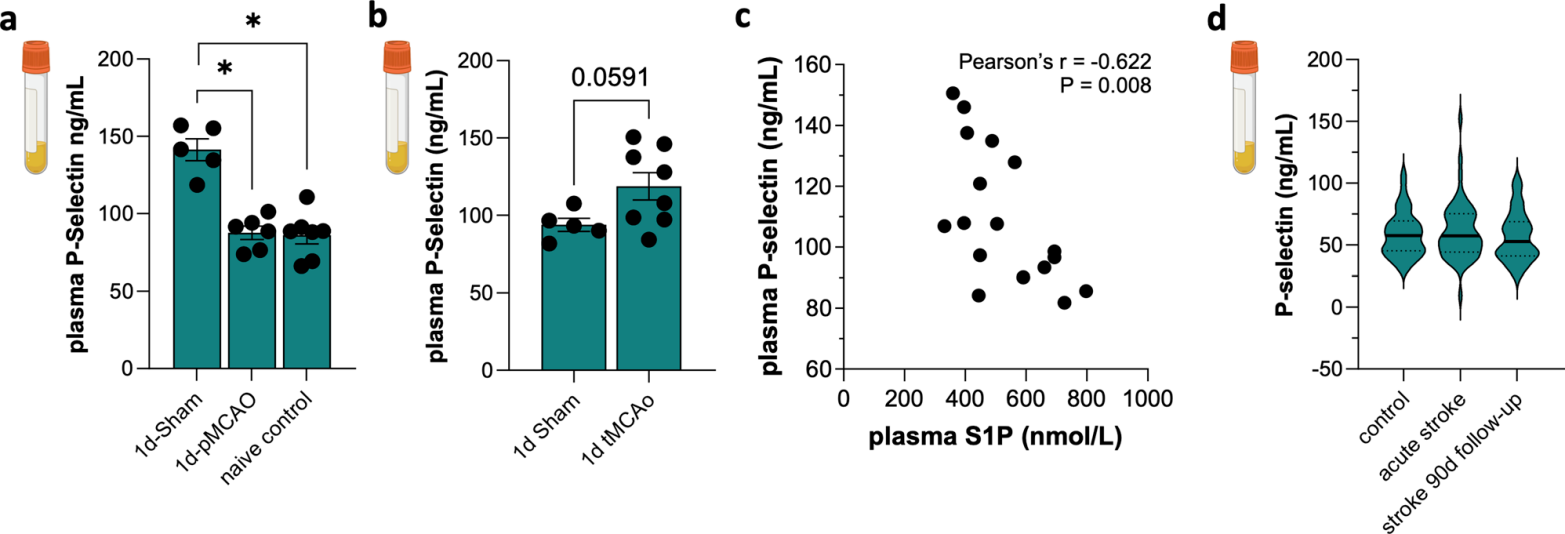
Plasma P-selectin levels after ischemic murine and human stroke. Plasma P-selectin concentrations measured in (**a**) mice 1-day after permanent middle cerebral artery occlusion (pMCAo; *N* = 6), in Sham controls (*N* = 5) or naïve mice (*N* = 7), and (**b**) in mice 1-day after transient middle cerebral artery occlusion (tMCAo; *N* = 8) and respective sham controls (*N* = 5). (**c**) Pearson correlation of plasma P-selectin and plasma sphingosine-1-phosphate (S1P) in mice 1-day after tMCAo and respective sham surgery. Correlation coefficient and calculated *p*-value are given. (**d**) Plasma P-selectin concentrations measured in healthy controls and in patients during the acute phase after ischemic stroke show no significant differences between groups. *N* = 47 for controls and *N* = 50 for ischemic stroke samples. Data are presented as mean ± SEM (**a, b**) or median ± interquartile range (**d**)

To further assess endothelial activation and BBB dysfunction, we examined the expression of specific endothelial cell markers [39]. Vascular endothelial-cadherin (VE-Cadherin) expression was significantly reduced in the ipsilateral compared to the contralateral hemisphere at 1-day but not 3-days post-stroke in the pMCAo model (Fig. 4a–b). Similarly, endothelial transcripts for occludin (*Ocln*), E-selectin (*Sele*) and P-selectin (*Selp*) (Fig. 4c–e) were significantly changed in the ipsilateral endothelium 1-day but not 3-day after tMCAo (Suppl. Fig. 2 g-h), while intercellular adhesion molecule 1 (*Icam1*) expression remained unchanged (Fig. 4f, Suppl. Fig. 2i), indicating that stroke is accompanied by parallel changes in endothelial barrier/activation markers alongside reduced plasma S1P. In parallel, we measured plasma P-selectin as a systemic marker reflecting endothelial and/or platelet activation in the context of altered S1P availability and signaling [40–42]. Plasma P‑selectin levels were higher 1-day after tMCAo compared to sham‑operated mice, whereas 1-day following pMCAo they were reduced relative to sham controls and unchanged compared to naïve reference levels (Fig. 5a–b). The different systemic P‑selectin responses suggest model‑specific coupling between endothelial dysfunction and circulating activation markers. In line with this, an inverse relationship between plasma S1P and plasma P‑selectin was observed in the tMCAo model, but not in the pMCAo model (Fig. 5c). In patients with acute ischemic stroke (*N* = 50) and compared to age- and sex-matched controls (*N* = 47), no significant differences in plasma P-selectin were observed between groups (acute stroke: 57.31 ± 31.10 ng/mL vs. control: 57.67 ± 24.15 ng/mL, *p* > 0.999; Fig. 5d). At 90-days post-stroke, P-selectin levels remained unchanged (Fig. 5d). However, we observed a significant inverse association between plasma S1P and P-selectin levels during the acute phase (Spearman ρ = −0.440, *p* = 0.001; Table 3), which was not present at follow-up (Spearman ρ = −0.131, *p* = 0.364). Collectively, these data indicate that acute post‑stroke alterations in S1P are associated with transient endothelial barrier dysfunction, with circulating P‑selectin reflecting context‑dependent vascular activation.

**Table 3:** Spearman correlations between plasma S1P and plasma P-selectin in ischemic stroke patients and controls

	Spearman ρ	P
Acute plasma S1P (nmol/mL)		
Plasma P-selectin (ng/mL)	−0.440	0.001
Follow-up plasma S1P (nmol/mL)		
Plasma P-selectin (ng/mL)	−0.131	0.364
Control plasma S1P (nmol/mL)		
Plasma P-selectin (ng/mL)	−0.182	0.211

Correlation analysis with Spearman coefficients. Stroke patients (*N* = 50, with 52 % female) collected acutely after the stroke event (i.e., within 11-days; median 56-hours) and a 90-days follow-up, and from control subjects (*N* = 47 with 51 % female)

**Fig. 6:**
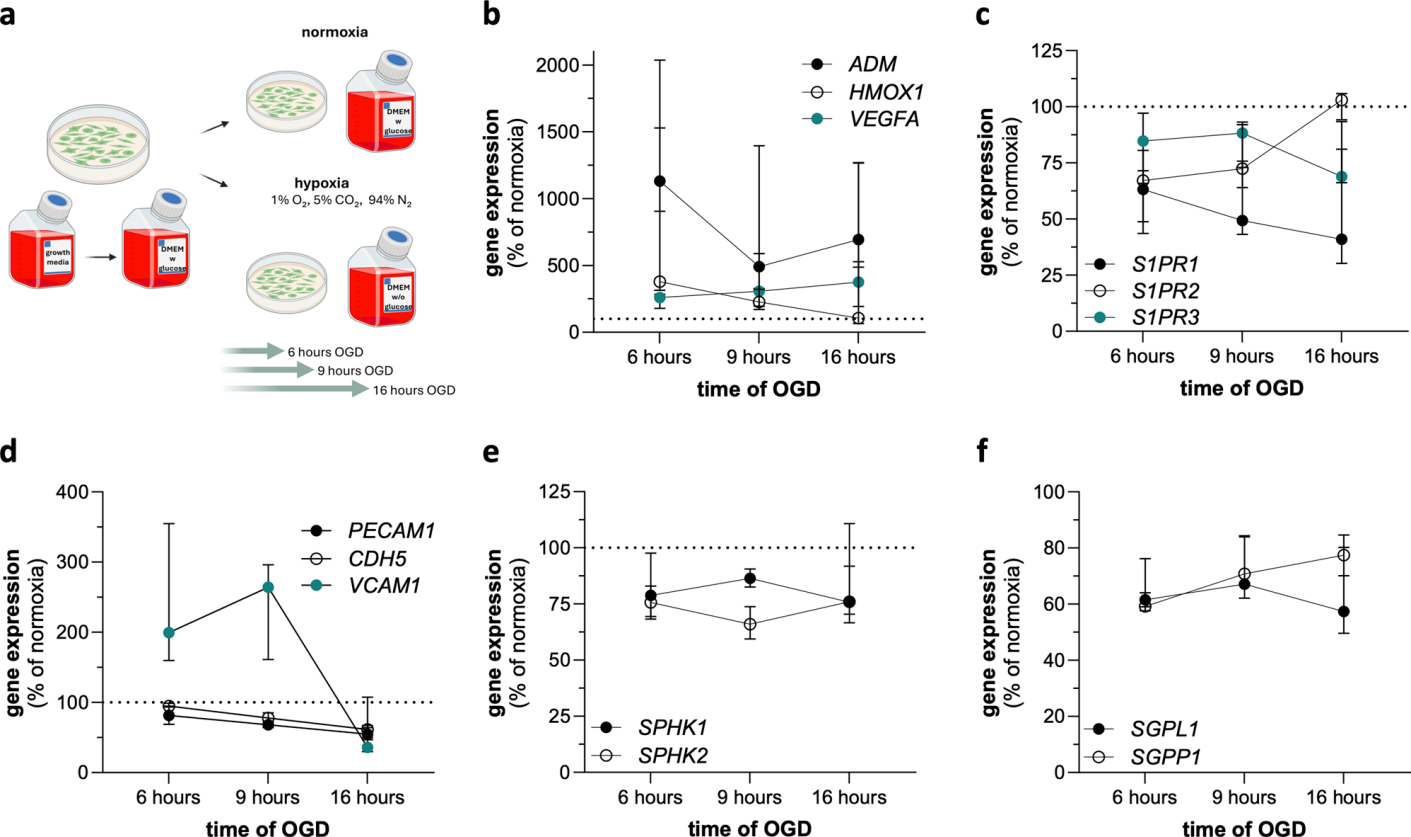
Regulation of endothelial gene expression by oxygen-glucose deprivation in human microvascular endothelial cells. (**a**) Schematic showing experimental design. (**b**) mRNA expression of hypoxia-responsive genes (*ADM*, *HMOX1*, and *VEGFA*) increases during oxygen-glucose deprivation (OGD). (**c**) Expression of S1P receptors (*S1PR1*-*3*) is reduced in a duration-dependent manner during OGD. (**d**) Expression of endothelial markers (*CDH5* and *PECAM1*) is reduced in a duration-dependent manner. *VCAM1* is increased at 6 h and 9 h of OGD. (**e**) Expression of *SPHK1* and *SPHK2* decreases with OGD exposure. (**f**) Expression of the S1P-degrading enzymes *SGPL1* and *SGPP1* is reduced with OGD. *N* = 9 for all readouts. Data are presented as percentage of normoxic controls and shown as median ± interquartile range. Dashed line represents 100 % of normoxia. *ADM – adrenomedullin, CDH5 – cadherin 5, HMOX1 – heme oxygenase 1, OGD – oxygen glucose deprivation, PECAM1 – platelet endothelial cell adhesion molecule-1, S1P – sphingosine-1-phosphate, S1PR – S1P receptor, SPHK – sphingosine kinase, SGPL – sphingosine lyase, SGPP – sphingosine phosphatase, VCAM1 – vascular cell adhesion molecule 1, VEGFA – vascular endothelial growth factor A*

To determine whether ischemia‑like conditions directly affect endothelial S1P signaling and endothelial integrity, independent of systemic influences, cultured human brain microvascular endothelial cells were exposed to increasing durations of oxygen-glucose deprivation (OGD; 1 % O_2_, 5 % CO_2_; 0 % Glucose; Fig. 6a). OGD induced a time-dependent upregulation of the hypoxia-responsive genes adrenomedullin (*ADM*), heme oxygenase-1 (*HMOX1*), and vascular endothelial growth factor A (*VEGFA*) (Fig. 6b; Suppl. Table 4), confirming a robust cellular response to hypoxic stress [43–45]. OGD profoundly altered endothelial S1P receptor expression. Under baseline conditions, *S1PR3* was the most abundantly expressed S1P receptor transcript (Suppl. Fig. 6a) and was consistently reduced across all OGD time points, while *S1PR1* expression also declines, with the strongest reduction after 16 h (Fig. 6c, Suppl. Table 4). *S1PR2* showed transient downregulation at 6 and 9 h of OGD, whereas *S1PR4* and *S1PR5* transcripts were below the detection limit (Fig. 6c, Suppl. Table 4). In cultured murine brain endothelial cells, *S1pr1* was the only consistently detectable receptor and showed an initial increase followed by a downregulation with prolonged OGD (Suppl. Fig. 7c). In parallel, OGD reduced expression of the endothelial marker genes *PECAM1* (platelet/endothelial cell adhesion molecule 1) and *CDH5* (cadherin 5) (Fig. 6d, Suppl. Table 4). Ischemic stress also affected S1P metabolism: under baseline conditions, *SPHK1* transcripts were more abundant than *SPHK2* in human endothelial cells, and OGD reduced expression of both enzymes, with preferential reduction of *SPHK2* at 9 h (Fig. 6e, Suppl. Table 4). In murine endothelial cells, baseline *Sphk2* expression was higher, but OGD induced a stronger reduction in *Sphk1* than in *Sphk2* (Suppl. Fig. 7e). Expression of the S1P-degrading enzymes *SGPP1* and *SGPL1* was likewise reduced under OGD, with strongest decrease in *SGPL1* observed after 16 h, while *SGPP2* transcripts were not detectable (Fig. 6f, Suppl. Table 4). Collectively, these in vitro data show that ischemia-like conditions directly suppress endothelial S1P receptor expression and S1P metabolic enzyme, with *SPHK2/Sphk2* representing the primary ischemia-responsive isoform and are accompanied by molecular features of endothelial activation consistent with the in vivo findings.

## Discussion

This study demonstrates a significant reduction in plasma S1P levels in patients with acute ischemic stroke compared with age- and sex-matched controls and is consistent with previous reports suggesting that S1P depletion is a hallmark of ischemic stroke and may contribute to secondary brain injury [22, 23]. In contrast to earlier work [22], plasma S1P did not correlate with initial stroke severity, 90-day functional outcome, or systemic inflammation in the present cohort, suggesting that although S1P depletion appears to be a robust feature of acute stroke, its relationship to clinical outcome is likely influenced by additional factors, including severity and previous cardiovascular events [22]. Multivariable logistic regression further showed that lower plasma S1P remained independently associated with acute ischemic stroke status after adjustment for relevant covariates, supporting the robustness of this association in the human cohort. Consistent with the human data, plasma S1P was also reduced in mice after both transient and permanent MCAo, indicating that reduced circulating S1P is a shared feature of experimental and clinical ischemic stroke.

The murine data further show that circulating S1P is differentially regulated in transient and permanent ischemia. In the tMCAo model, plasma S1P fell below both sham and naïve levels, whereas in the pMCAo model it was reduced relative to sham-operated animals but remained within the naïve range. Together with the effect of sham surgery itself on plasma S1P, this indicates that circulating S1P in the experimental setting integrates stroke-related injury with procedure-related systemic confounders such as anesthesia, vascular manipulation, tissue trauma, and stress responses, and cannot be interpreted as an endothelial-specific readout. This is particularly relevant in view of the multiple non-endothelial sources of circulating S1P, including erythrocytes, platelets, and HDL-associated plasma pools [24, 25], and the prominent role of platelet activation in ischemia/reperfusion injury after tMCAo [46]. Nevertheless, the cerebral endothelium emerged as a stroke-responsive compartment with clear alterations in S1P pathway regulation, although the circulating plasma S1P changes observed here cannot be assigned specifically to endothelial sources.

Endothelial-specific RiboTag analysis revealed acute downregulation of endothelial *Sphk1* and *Sphk2* expression after tMCAo, and additional protein analyses showed reduced endothelial SPHK2 abundance in isolated cerebral vessels after pMCAo and a lowering of SPHK expression in human endothelial cells after OGD. These findings strengthen the evidence that ischemic stress is accompanied by suppression of endothelial S1P synthesis-related components at both the transcript and protein level. Previous studies have established distinct roles for SphK1 and SphK2 in the generation of intracellular and circulating S1P pools [13, 47, 48]. SphK1 is generally considered a major endothelial isoform supporting stimulus-responsive S1P production and vascular homeostasis [13, 25], whereas SphK2 contributes more substantially to circulating S1P through erythrocytes, platelets, and other non-endothelial sources [49–51]. In our datasets, both isoforms were suppressed after ischemic stress, with model-dependent differences between in vivo and in vitro systems. In addition to S1P generation, S1P degradation contributes to the plasma S1P pool [24, 25]. In parallel to SphKs, expression of the major S1P-degrading enzymes *Sgpl1*, *Sgpp1*, and *Sgpp2* was also reduced after ischemic stroke, indicating a broad dysregulation of endothelial S1P metabolic pathways rather than a selective suppression of synthesis alone. As these observations are based primarily on transcriptomic profiling, they should be interpreted as descriptive evidence of S1P pathway dysregulation rather than functional proof of altered endothelial S1P metabolism, the direction of net S1P flux after stroke, or an endothelial-specific contribution to the observed plasma S1P lowering post-stroke.

Disruption of S1P signaling has been suggested as an indicator of endothelial activation [14, 52, 53], supported by upregulation of adhesion molecules such as P-selectin and E-selectin [40, 52, 54], both of which facilitate leukocyte adhesion and contribute to post-stroke inflammation and BBB disruption [55–57]. In line with this, experimental stroke was accompanied by coordinated changes in endothelial barrier- and activation-related markers [40, 52, 54], with reduced VE-cadherin after pMCAo and altered endothelial *Ocln*, *Sele*, and *Selp* expression after tMCAo. To extend these tissue-level findings to the circulation, we assessed plasma P-selectin as a systemic readout related to endothelial and/or platelet activation. The corresponding data point to context-dependent deregulation rather than a uniform endothelial signature across models. In the human cohort, plasma P-selectin was not elevated at the group level, yet it showed a significant inverse association with plasma S1P during the acute phase. In the murine models, plasma P-selectin increased after tMCAo but not after pMCAo, where levels were lower than in sham-operated mice and like naïve controls. Thus, a pattern consistent with an inverse relationship between circulating S1P and P-selectin was present in transient/reperfused ischemia, but not in permanent cortical ischemia in the experimental settings. Because stroke subtype and reperfusion status were not resolved in the human cohort, this distinction cannot be made directly in the clinical data. Nevertheless, the combined findings indicate that the relationship between circulating S1P and P-selectin is context-dependent and may be particularly pronounced under conditions of acute reperfusion-associated endothelial and platelet activation [46, 58] but should not be interpreted as a uniform marker of post-stroke endothelial dysfunction.

The S1P receptor data likewise support impaired endothelial S1P signaling as a feature of stroke. A key function of endothelial-derived S1P is to promote vascular homeostasis by regulating vascular tone [4, 10], endothelial barrier integrity [17, 37, 38, 59] and modulating inflammation [37, 59–62] mainly through the activation of S1PR1, which enhances the integrity of the endothelial barrier and limits vascular leakage [17, 59, 63]. In line with this, expression of *S1pr1*, *S1pr3*, and *S1pr4* was reduced in the ischemic hemisphere, in parallel with the observed changes in endothelial barrier- and activation-related markers. Given the central role of S1PR1 in preserving endothelial junctional integrity [37, 64–66], the association between lower cerebrovascular S1PR1 protein expression and greater parenchymal serum albumin accumulation is consistent with reduced barrier-supportive S1P signaling after stroke. *S1pr4*, although expressed at lower levels, was also reduced in murine ischemic endothelium. Prior work indicates that even lower-abundance receptors such as S1PR4 can influence barrier properties in brain microvessels [38, 67], suggesting that suppression of both *S1pr1* and *S1pr4* may contribute to endothelial destabilization. In contrast, *S1pr2* was below the detection threshold in the murine endothelial datasets but detectable in cultured human brain microvascular endothelial cells. Although expression below the detection threshold does not rule out the possibility of low-level or context-dependent S1PR2/*S1pr2* expression in other endothelial systems, the differences between the murine in vivo and human in vitro receptor profiles likely reflect species-related differences and the distinct experimental systems used. Endothelial RiboTag isolates were obtained from ischemic mouse brain within the intact neurovascular unit, where endothelial cells remain exposed to blood flow, circulating S1P carriers, reperfusion-associated hemodynamic changes, and paracrine signals from pericytes, astrocytes, neurons, and infiltrating immune cells. In contrast, the OGD experiments were performed in immortalized human brain microvascular endothelial cells in monoculture and therefore model a cell-autonomous response to hypoxia/glucose deprivation in the absence of these vascular and tissue-level cues. This difference in context likely contributes to the observed divergence in baseline receptor composition and ischemic responsiveness. Nonetheless, in both systems ischemic stress was associated with reduced endothelial S1P pathway signaling capacity. Given the beneficial effects of S1PR modulators in clinical trials [68–70], targeting S1P signaling could represent a promising therapeutic avenue for mitigating post-stroke endothelial dysfunction. Future studies should investigate whether pharmacological restoration of S1P levels can improve BBB integrity and enhance functional recovery in ischemic stroke by boosting cerebrovascular S1PR signaling.

## Conclusions

This study shows that acute ischemic stroke is associated with reduced plasma S1P levels, altered brain-endothelial S1P pathway expression, signs of endothelial activation, and BBB disruption. Together, these findings support plasma S1P as a candidate circulating marker associated with cerebrovascular injury after ischemic stroke. Further work is required to clarify the mechanistic basis of these associations and to determine whether targeting the S1P signaling axis may have therapeutic relevance in ischemic stroke.

### Limitations

Several limitations should be considered when interpreting the present findings. Foremost, the study identifies parallel changes in circulating plasma S1P, endothelial S1P pathway expression, endothelial activation-related marker expression, and BBB disruption following stroke; however, it does not establish a direct mechanistic link between these processes. Notably, the absence of S1P restoration experiments, endothelial-specific SPHK manipulation, and pharmacologic S1PR1 rescue precludes drawing conclusions about causality. Additionally, the analyses primarily focused on transcript regulation and selected protein validation, rather than directly assessing enzyme activity or S1P metabolic flux through isotope tracing or labeling approaches. Thus, although the data support the notion that the endothelial S1P pathway is regulated differently after stroke, they do not define the functional contribution of individual pathway components. Furthermore, circulating plasma S1P reflects multiple sources, including endothelial cells, erythrocytes, platelets, and HDL-associated plasma pools. Since the present study did not distinguish between these compartments, the relative contribution of endothelial versus non-endothelial sources to the observed plasma S1P reduction remains unknown. Directly assessing erythrocyte-, platelet-, and HDL-associated S1P would require source-specific lipid analyses, which lies beyond the scope of this study.

Additional limitations apply to the human cohort and the experimental models. The human cohort was relatively small and consisted predominantly of patients with mild stroke, which may limit the ability to generalize to more severe ischemic injury. Although multivariable analysis supported an independent association between lower plasma S1P levels and acute stroke status, the observational design does not permit inference of causality. In the experimental studies, only male mice were used, which precludes the assessment of sex-specific differences. Additionally, formal a priori power calculations were not performed. In the murine models, sham surgery itself influenced circulating S1P and P-selectin levels, indicating that these plasma readouts reflect integrated systemic responses to ischemic injury, surgical stress, and non-endothelial circulating sources rather than a purely cerebral endothelial signal. Finally, since the in vitro and in vivo models capture distinct biological contexts, differences between murine endothelial translatome analyses and cultured human endothelial cells should be interpreted cautiously.

## Electronic supplementary material

Below is the link to the electronic supplementary material.

Supplementary Material 1

Supplementary Material 2

## Data Availability

All data generated or analyzed during this study are included in this published article and its supplementary information files.

## Abbreviations

Adm: Adrenomedullin
AHT: Anti-hypertensive treatment
BBB: Blood-brain barrier
BMI: Body mass index
BP: Blood pressure
CCA: Common carotid artery
Cdh5: Cadherin 5
CRP: C-reactive protein
ECA: External carotid artery
HBMECs: Human brain microvascular endothelial cells
HDL: High-density lipoprotein
Hmox1: Heme oxygenase-1
ICA: Internal carotid artery
Icam1: Intercellular adhesion molecule 1
MCA: Middle cerebral artery
MCAo: Middle cerebral artery occlusion
mRS: Modified Rankin Scale
NIHSS: National Institutes of Health Stroke Scale
Ocln: Occludin
OGD: Oxygen glucose deprivation
Pecam1: Platelet/endothelial cell adhesion molecule 1
pMCAo: Permanent middle cerebral artery occlusion
S1P: Sphingosine-1-phosphate
S1PR: Sphingosine-1-phosphate receptor
Sele: E-selectin
Selp: P-selectin
Sgpl1: Sphingosine-1-phosphate lyase 1
Sgpp1: Sphingosine-1-phosphate phosphatase 1
Sgpp2: Sphingosine-1-phosphate phosphatase 2
Sphk1: Sphingosine kinase 1
Sphk2: Sphingosine kinase 2
tMCAO: Transient middle cerebral artery occlusion
VE-Cadherin: Vascular endothelial-cadherin
Vegfa: Vascular endothelial growth factor A
