# Supplementary material for: Circulating sphingosine-1-phosphate depletion is associated with endothelial activation and altered brain-endothelial S1P pathway expression in ischemic stroke
Source: Fluids Barriers CNS. 2026 Jun 12;23:82. doi: 10.1186/s12987-026-00828-z (PMC13285081; doi:10.1186/s12987-026-00828-z)
Supplement: Supplementary file 1 — Supplementary Material 1 [file 12987_2026_828_MOESM1_ESM.docx]

**Circulating Sphingosine-1-Phosphate Depletion Is Associated with Endothelial Activation and Altered Brain-Endothelial S1P Pathway Expression in Ischemic Stroke**

Lisa T Porschen^a,b,c^, Frank Matthes^a,b,c^, Hana Matuskova^a,b,e,f^, Lino Braadt^c,d^, Gabor C Petzold^e,f^, Arne G Lindgren^g,h^ & Anja Meissner^a,b,c^

^a^ Department of Experimental Medical Sciences, Lund University, 221 84 Lund, Sweden

^b^ Wallenberg Centre for Molecular Medicine, Lund University, 221 84 Lund, Sweden

^c^ Division of Physiology & Vascular Biology, Institute of Theoretical Medicine, Faculty of Medicine, University of Augsburg, Augsburg, Germany

^d^ Department of Vascular Neurology, University Hospital Bonn, 53127 Bonn, Germany

^e^ German Center for Neurodegenerative Diseases (DZNE), 53127 Bonn, Germany

^f^ Department of Neurology and Clinical Neurophysiology, University Hospital Augsburg, Augsburg, Germany

^g^ Department of Clinical Sciences Lund, Neurology, Lund University, Lund, Sweden

^h^ Department of Neurology, Rehabilitation Medicine, Memory Disorders and Geriatrics, Skåne University Hospital, Lund, Sweden

*
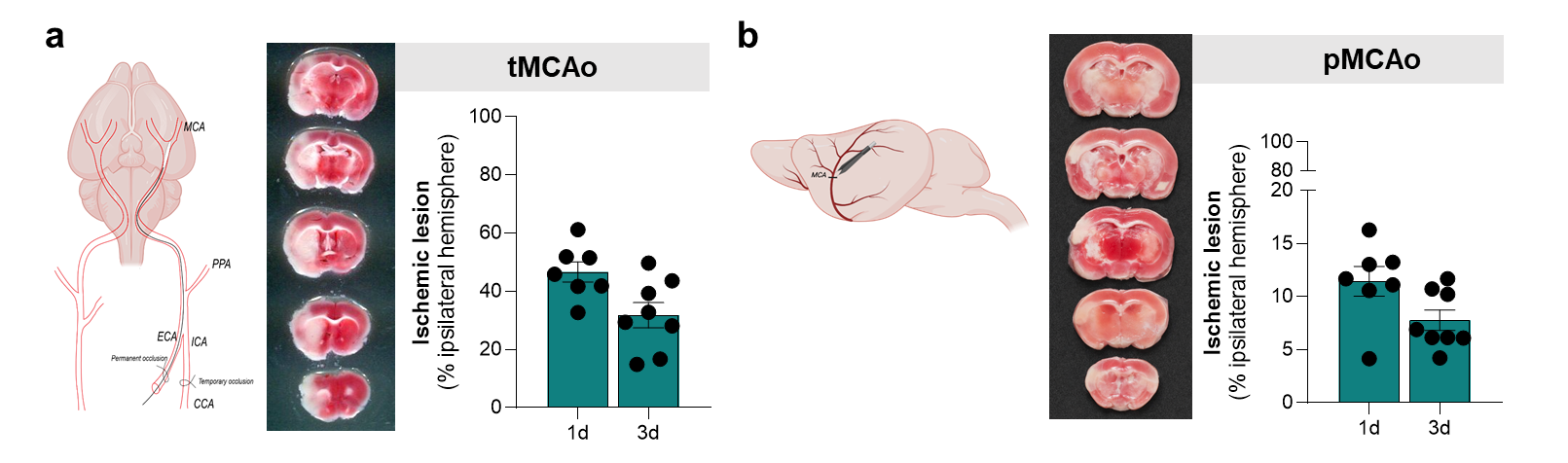
*

**Suppl. Figure 1. Characterization of the experimental stroke models.**

**(a)** Schematic of transient middle cerebral artery occlusion (tMCAo). tMCAo was induced by intraluminal filament occlusion of the MCA for 60 min followed by reperfusion. Representative TTC-stained brain sections and quantification of infarct size at 1- and 3-days post-stroke are shown. **(b)** Schematic of permanent middle cerebral artery occlusion (pMCAo). pMCAo was induced by permanent electrocoagulation and transection of the MCA. Representative TTC-stained brain sections and quantification of infarct size at 1- and 3-days post-stroke are shown. *CCA – common carotid artery, ECA – external carotid artery, ICA – internal carotid artery, MCA – middle cerebral artery, pMCAo – permanent middle cerebral artery occlusion, tMCAo – transient middle cerebral artery occlusion.*


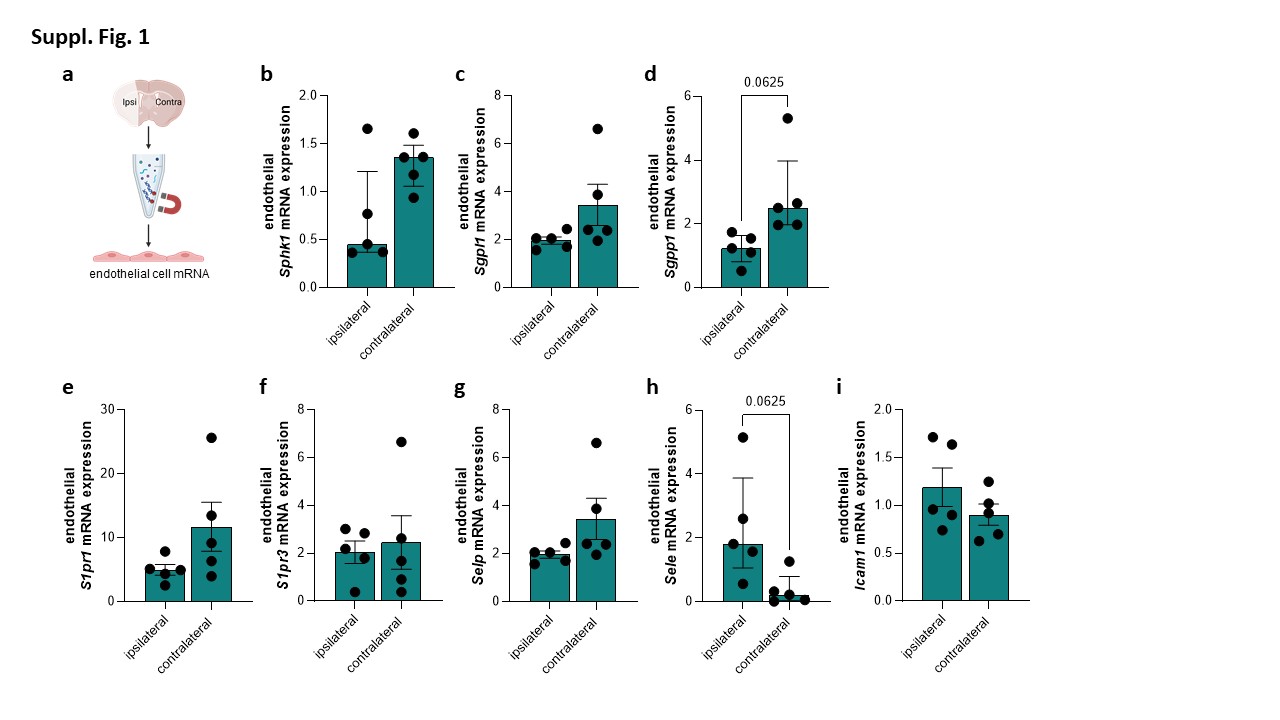


**Suppl. Figure 2. Endothelial expression of S1P signaling components 3-days after transient middle cerebral artery occlusion.**

**(a)** Schematic overview of endothelial-specific mRNA isolation using the RiboTag approach from cerebral endothelial cells 3-days after transient middle cerebral artery occlusion (tMCAo).

Endothelial mRNA expression of sphingosine kinase 1 (*Sphk1*) **(b)**, sphingosine-1-phosphate lyase (*Sgpl1*) **(c),** sphingosine-1-phosphate phosphatase 1 (*Sgpp1*) **(d),** sphingosine-1-phosphate receptor 1 (*S1pr1*) **(e),** and sphingosine-1-phosphate receptor 3 (*S1pr3*) **(f)**, P-selectin (*Selp*) **(g)**, E-selectin (*Sele*) **(h)** and intercellular adhesion molecule 1 (*Icam1*) **(i)** shows no significant differences between the ischemic and contralateral hemispheres. N = 5 for all readouts. Data are presented as mean ± SEM **(c, e-g, i)** or median ± interquartile range **(b, d, h)**. *Contra – contralateral, intercellular adhesion molecule 1 (Icam1), ipsi – ipsilateral,* *Sele – E-selectin, Selp – P-selectin,* *S1P – sphingosine-1-phosphate, Sphk – sphingosine kinase, Sgpl – sphingosine lyase, Sgpp – sphingosine phosphatase.*


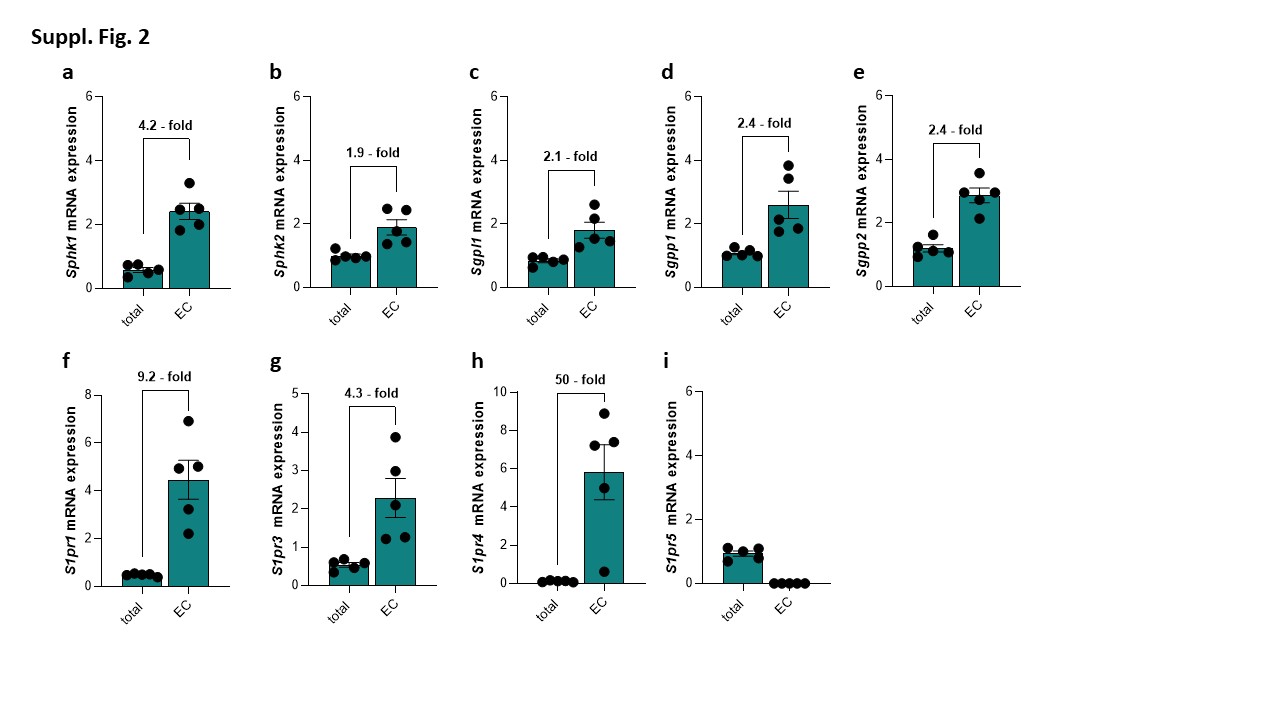


**Suppl. Figure 3. Endothelial enrichment of S1P signaling components.**

**(a–i)** Endothelial mRNA isolated from RiboTag mice shows significant enrichment of multiple S1P-signaling components compared with total tissue homogenates, indicating predominant endothelial expression. **(j)** *S1pr5* shows no endothelial enrichment. N = 5 for all readouts. Data are presented as mean ± SEM. *EC - endothelial cells;* *S1P – sphingosine-1-phosphate,* *S1pr – S1P receptor, Sphk – sphingosine kinase, Sgpl – sphingosine lyase, Sgpp – sphingosine phosphatase.*

*
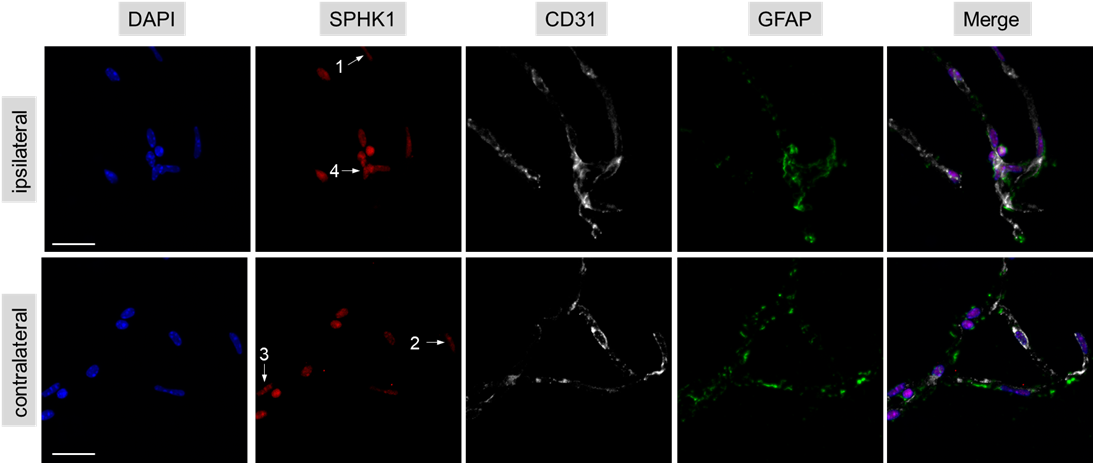
*

**Suppl. Figure 4. Endothelial expression of SPHK1 in isolated cerebral vessels 1-day post-permanent middle cerebral artery occlusion.**

Representative immunofluorescence images of cerebral vessels isolated 1-day after permanent middle cerebral artery occlusion (pMCAo) stained for 4′,6-diamidino-2-phenylindole (DAPI), cluster of differentiation 31 (CD31), glial fibrillary acidic protein (GFAP) and sphingosine kinase 1 (SPHK1). Arrows are indicating scoring examples for SPHK1 intensity score (score: 0 = no detectable signal, 1 = low, 2 = moderate, 3 = strong, 4 = very strong). *Cluster of differentiation 31* *– CD31, GFAP – glial fibrillary acidic protein, Sphk – sphingosine kinase.*


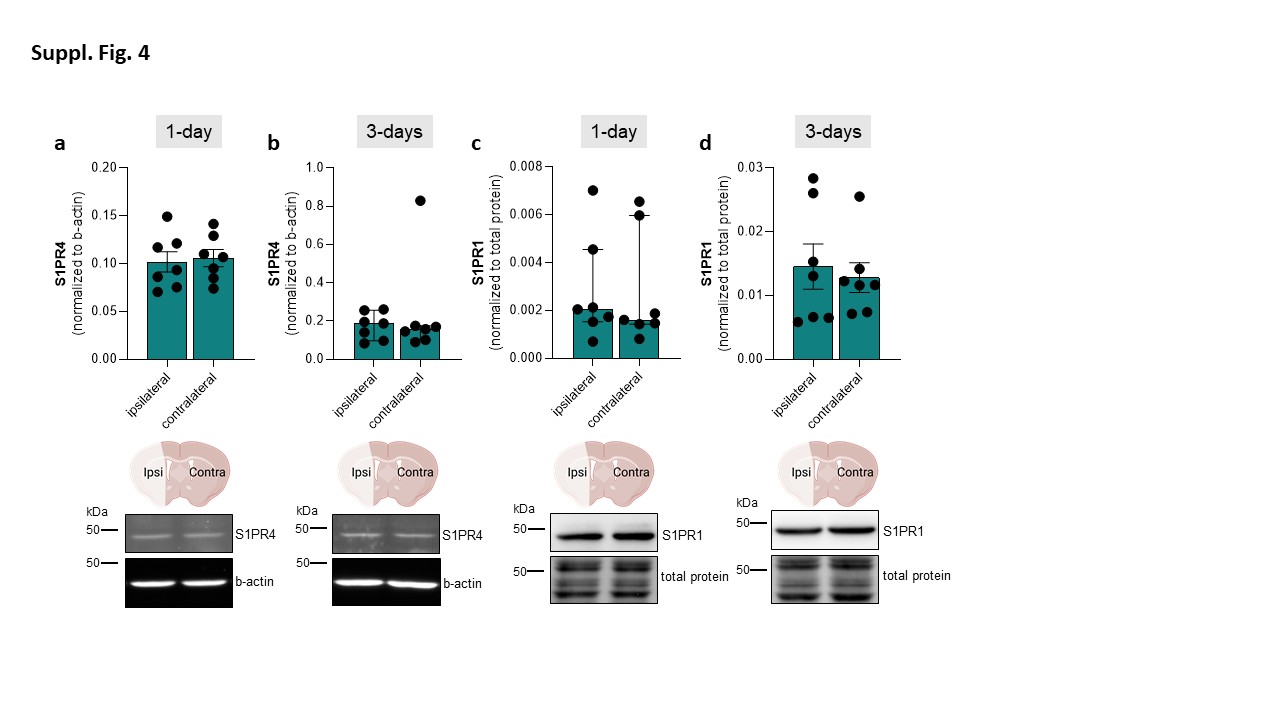


**Suppl. Figure 5. S1P receptor protein expression in whole-hemisphere homogenates after permanent ischemic stroke.**

Protein expression levels of S1PR4 at 1-day **(a)** and 3-days **(b)** after permanent middle cerebral artery occlusion (pMCAo), and S1PR1 at 1-day **(c)** and 3-days **(d)** after pMCAo, measured in whole-hemisphere homogenates. Representative immunoblots are shown for each condition. No significant differences were detected between ischemic and contralateral hemispheres at either time point. N = 7 for all readouts. Data are presented as mean ± SEM **(a, d)** or median ± interquartile range **(b, c)**. *Contra – contralateral, ipsi – ipsilateral, S1P – sphingosine-1-phosphate, S1pr – S1P receptor.*


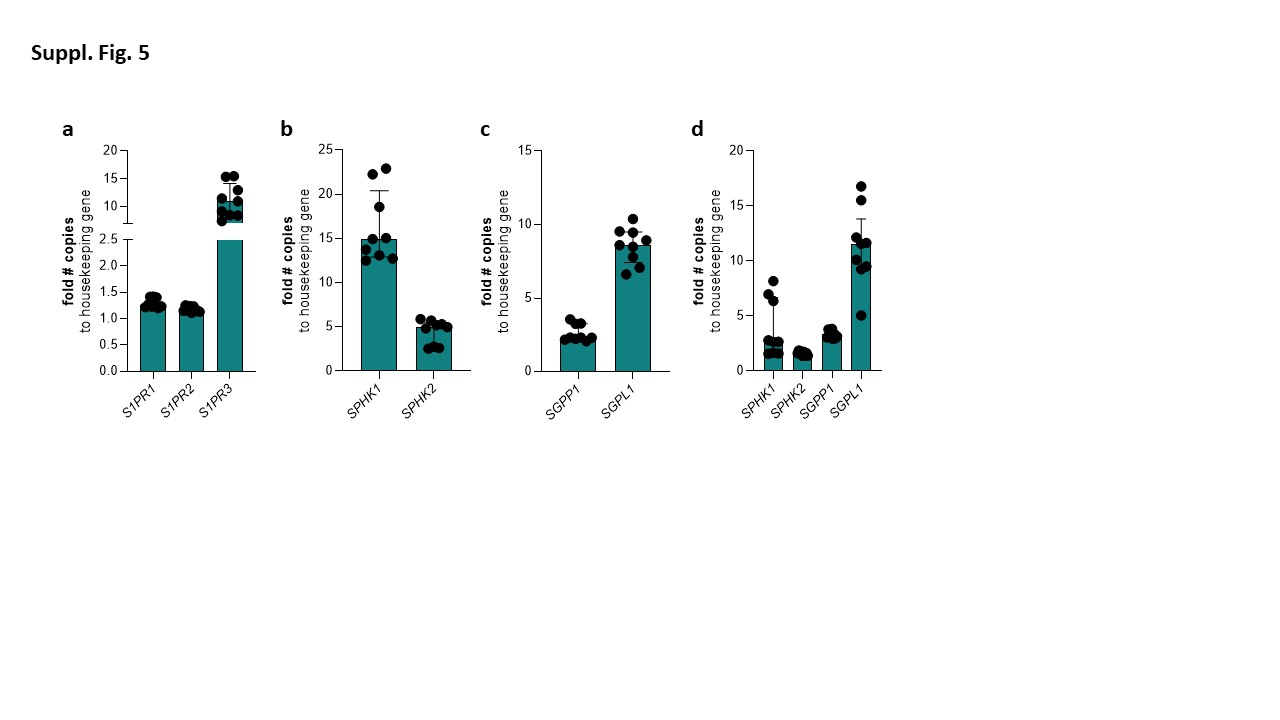


**Suppl. Figure 6. Absolute mRNA expression of S1P signaling components in human microvascular brain endothelial cells.**

**(a)** Absolute mRNA expression of sphingosine-1-phosphate receptors (*S1PR1–3*). **(b)** Absolute mRNA expression levels of sphingosine kinases (*SPHK1* and *SPHK2*). **(c)** Absolute mRNA expression of sphingosine-1-phosphate degrading enzymes, including sphingosine-1-phosphate lyase (*SGPL1*) and sphingosine-1-phosphate phosphatase 1 (*SGPP1*). **(d)** Comparative analysis of absolute expression levels of all S1P metabolic enzymes. N = 9 for all readouts. Data are presented as mean ± SEM **(d)** or median ± interquartile range **(a-c)**. *S1P – sphingosine-1-phosphate, S1PR – S1P receptor, SPHK – sphingosine kinase, SGPL – sphingosine lyase, SGPP – sphingosine phosphatase.*


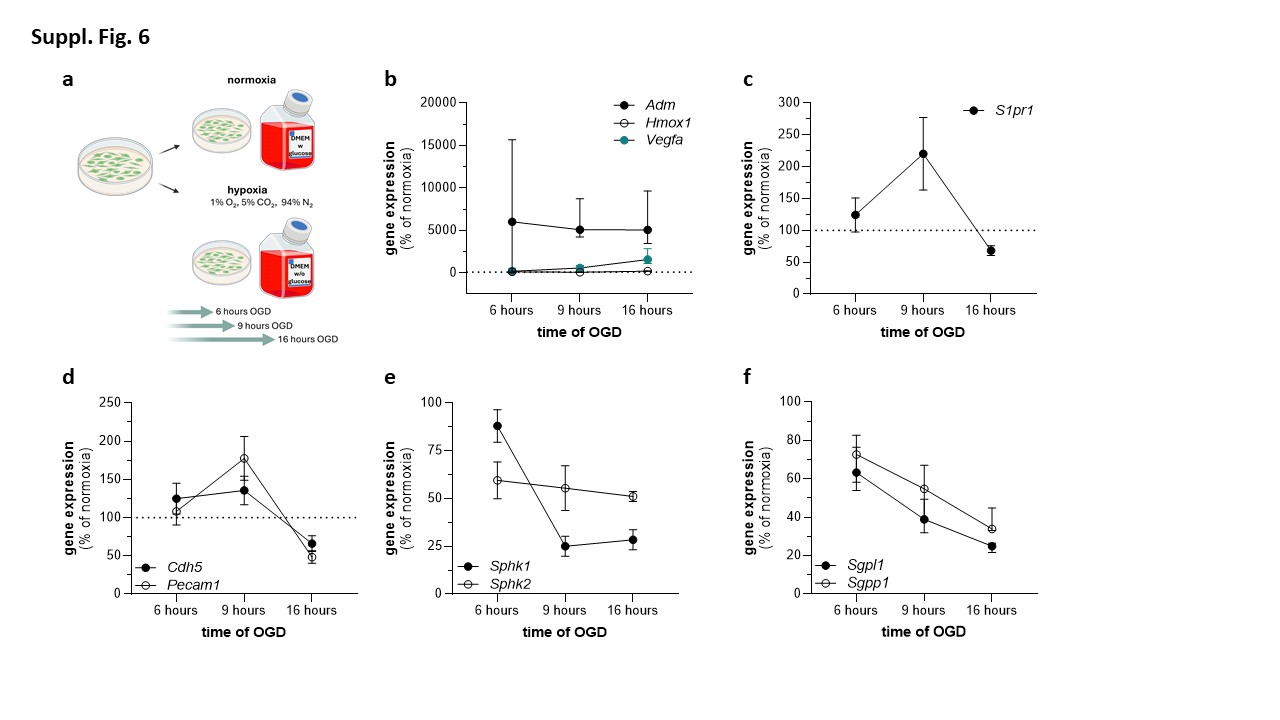


**Suppl. Figure 7. Regulation of endothelial gene expression by oxygen-glucose deprivation in murine brain endothelial cells.**

**(a**) Schematic showing experimental design. **(b)** mRNA expression of hypoxia-responsive genes (*Adm*, *Hmox1*, and *Vegfa*) is increased at all timepoints of oxygen–glucose deprivation (OGD). **(c)** Expression of sphingosine-1-phosphate receptor (*S1pr1*) is altered in a duration-dependent manner during OGD. **(d)** Expression of endothelial markers (*Cdh5* and *Pecam1*) is reduced at 16 hours of OGD. **(e)** Expression of sphingosine kinases *Sphk1* and *Sphk2* is reduced with increasing duration of OGD. **(f)** Expression of the S1P-degrading enzymes *Sgpl1* and *Sgpp1* progressively decreases with OGD exposure. N = 5-6 for all readouts. Data are presented as percentage of normoxic controls and shown as mean ± SEM **(c, d, e)** or median ± interquartile range **(b, f)**. Dashed line represents 100 % of normoxia. *Adm – Adrenomedullin*, *Cdh5 – cadherin 5, Hmox1 – heme oxygenase 1,* *OGD – oxygen glucose deprivation, Pecam1 – platelet endothelial cell adhesion molecule-1*, *S1P – sphingosine-1-phosphate, S1pr – S1P receptor, Sphk – sphingosine kinase, Sgpl – sphingosine lyase, Sgpp – sphingosine phosphatase, Vegfa – vascular endothelial growth factor A.*


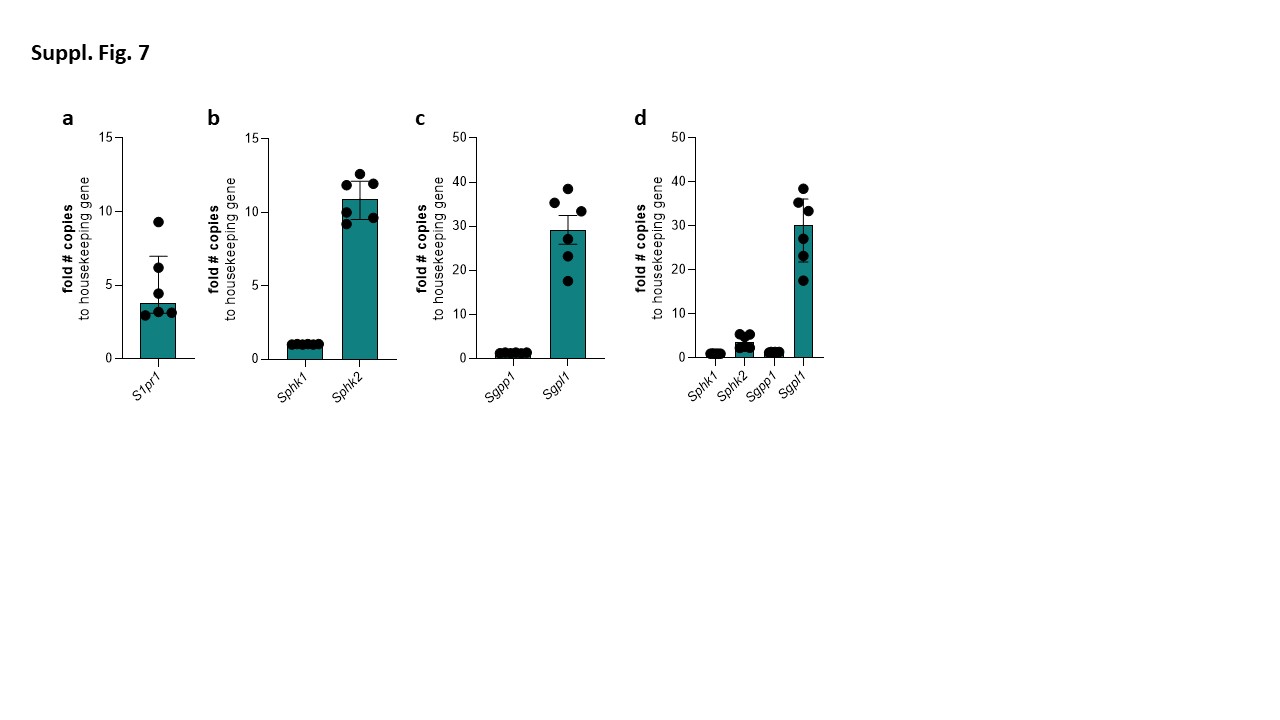


**Suppl. Figure 8. Absolute mRNA expression of S1P signaling components in murine brain endothelial cells.**

**(a)** Absolute mRNA expression of sphingosine-1-phosphate receptor (*S1pr1*). **(b)** Absolute mRNA expression levels of sphingosine kinases (*Sphk1* and *Sphk2*). **(c)** Absolute mRNA expression of the degrading enzymes sphingosine-1-phosphate phosphate lyase (*Sgpl1*) and sphingosine-1-phosphate phosphatase 1 (*Sgpp1*). **(d)** Comparative analysis of absolute expression levels of all S1P metabolic enzymes. N = 6 for all readouts. Data are presented as mean ± SEM **(c)** or median ± interquartile range **(a, b, d)**. *S1P – sphingosine-1-phosphate, S1pr* *– S1P receptor, Sphk – sphingosine kinase, Sgpl – sphingosine lyase, Sgpp – sphingosine phosphatase.*

**Suppl. Table 1. Key resources table for primer pairs used in qPCR.**

1. **Mouse**

| Target gene | Forward primer (5´→ 3’) | Reverse primer (5´→ 3’) |
| --- | --- | --- |
| *Rpl14* | GGCTTTAGTGGATGGACCCT | ATTGATATCCGCCTTCTCCC |
| *Sphk1* | GGTGAATGGGCTAATGGAACG | CTGCTCGTACCCAGCATAGTG |
| *Sphk2* | CACGGCGAGTTTGGTTCCTA | CTTCTGGCTTTGGGCGTAGT |
| *Sgpp1* | GAGCAACTTGCCGCTCTACTA | GGTCGAGATTCCAGATCCAGAA |
| *Sgpp2* | GTTCTCTACGCTGGTGTGTCT | GCAGGGTAGGTCAGAGCAAT |
| *Sgpl1* | GGTGTATGAGCTTATCTTCCAGC | CTGTTGTTCGATCTTACGTCCA |
| *S1pr1* | ATGGTGTCCACTAGCATCCC | CGATGTTCAACTTGCCTGTGTAG |
| *S1pr2* | ACAGTCTCCAAAACCAACCACT | ACTCTGAGTATAAGCCGCCCA |
| *S1pr3* | CAAGCCTAGCGGGAGAGAAA | ACTGCGGGAAGAGTGTTGAA |
| *S1pr4* | CTCGGTCACTCAGCTTCAGG | CAGTCACCATCAGGCAAGGT |
| *S1pr5* | GCCTAGGGCACACGACC | GACACAGGAGAGCGGCTATT |
| *Selp* | CATCTGGTTCAGTGCTTTGATCT | ACCCGTGAGTTATTCCATGAGT |
| *Sele* | CACGACATGCACGTTTGACT | GGCGCAGATAAGGCTTCACA |
| *Icam1* | CCGTGCCAAGCCCACGCTAC | GCGGAAGCGGACGACTGCAC |
| *Vcam1* | TGCCGAGCTAAATTACACATTG | CCTTGTGGAGGGATGTACAGA |
| *Pecam1* | CCAAAGCCAGTAGCATCATGGTC | GGATGGTGAAGTTGGCTACAGGG |
| *Cdh5* | ATGGCAGGCCCTAACTTTCC | CATTCTGGCGGTTCACGTTG |
| *Vegfa* | GGCCTCCGAAACCATGAACT | GTCCACCAGGGTCTCAATCG |
| *Adm* | TTCTCGGCTTCTCATCGCAG | TCAGGGTGATGGAAACCAGC |
| *Hmox1* | GAATCGAGCAGAACCAGCCT | CTCAGCATTCTCGGCTTGGA |
| *Ocln* | GAAAGGGCAAGAGGACGGAC | CGTCGTCTAGTTCTGCCTGT |

1. **Human**

| Target gene | Forward primer (5´→ 3’) | Reverse primer (5´→ 3’) |
| --- | --- | --- |
| *GPI* | AGGCTGCTGCCACATAAGGT | CCAAGGCTCCAAGCATGAAT |
| *SPHK1* | AGGCTGAAATCTCCTTCACGC | GTCTCCAGACATGACCACCAG |
| *SPHK2* | CTGTCTGCTCCGAGGACTGC | CAAAGGGATTGACCAATAGAAGC |
| *SGPP1* | CACCGCCATCCCCATTTCTA | AACTAGAGAACACCAGCAGGG |
| *SGPP2* | CCATTGCCTTCACCCTCCTT | CCAGTGTAGAGCCTGCTGAG |
| *SGPL1* | ACTGCTCGCTTCCTCAAGTC | TACTGGGTGGGAACTGCAAC |
| *S1PR1* | TCTGCGGGAAGGGAGTATGT | CGATGGCGAGGAGACTGAA |
| *S1PR2* | CCTTCTCTGGGCAACTTCGT | GGAGAAGTCGTCAAGTGGCA |
| *S1PR3* | TCTACCATCCTGCCCCTCTAC | ACACGCTCACCACAATCACC |
| *S1PR4* | CAACCCCATCATCTACTCCTTCC | AAAGCTGTCCCTTGGCCTC |
| *S1PR5* | TGTATTTATGCAGCGACACCCC | CTTCCCTGACCACCATCACC |
| *VCAM1* | CATCCGAAAGGCCCAGTTGA | TAGAGCACGAGAAGCTCAGGA |
| *PECAM1* | AAGTGGAGTCCAGCCGCATATC | ATGGAGCAGGACAGGTTCAGTC |
| *CDH5* | CGGCGCCAAAAGAGAGATTG | CGTCTCCTGTCTCTGCATCG |
| *VEGFA* | AAGGAGGAGGGCAGAATCATC | TCAGGGTACTCCTGGAAGATGT |
| *ADM* | CTTCGGAGTTTTGCCATTGCC | CTGGACATCCGCAGTTCCC |
| *HMOX1* | AACTTTCAGAAGGGCCAGGT | CTCCTCCAGGGCCACATAGA |

**Suppl. Table 2. Plasma sphingosine-1-phosphate (S1P) levels in transient and permanent experimental stroke models.**

Plasma S1P concentrations are shown for naïve mice and for sham- and MCAo-operated mice at either 1 or 3 days after transient or permanent middle cerebral artery occlusion. Data are presented as mean ± SEM for the transient model and median ± interquartile range for the permanent model. The same naïve reference group (N = 10) is shown for comparison with both stroke models. P-values are reported for multiple comparison testing using 1-Way ANOVA for the transient model and Kruskal-Wallis for the permanent model; significant post-hoc differences are indicated with * for MCAo and their respective sham groups and with & for 1d sham and naïve comparisons. *MCAo – middle cerebral artery occlusion, SEM – standard error of the mean, S1P – sphingosine-1-phosphate.*

| **occlusion type** | **1d Sham** | **1d MCAo** | **3d Sham** | **3d MCAo** | **naïve** | **P-value** |
| --- | --- | --- | --- | --- | --- | --- |
| permanent | 592.7 ± 126.4 | 489.1 ± 103.6* | 468.0 ± 65.0 | 440.0 ± 163.2 | 482.5 ± 114.8^&^ | 0.0234 |
| transient | 540.0 ± 58.6 | 370.8 ± 29.7* | 358.4 ± 26.8 | 482.1 ± 54.4 | 461.0 ± 23.8 | 0.0340 |

**Suppl. Table 3. mRNA expression of S1P signaling components in endothelial and total tissue of Cdh5^Cre-ER(T) mice 1-day post stroke.**

N = 5 for all readouts. Data are presented as mean $\pm$ SD. P-values given for paired comparisons. *S1P – sphingosine-1-phosphate, S1pr – S1P receptor, Sphk – sphingosine kinase, Sgpl – sphingosine lyase, Sgpp – sphingosine phosphatase*.

| Target gene | **endothelial cells** | | | | **total tissue** | | | |
| --- | --- | --- | --- | --- | --- | --- | --- | --- |
|  | ipsilateral | contralateral | P-value | adjusted P-value | ipsilateral | contralateral | P-value | adjusted P-value |
| S1P generating enzymes | | | | | | | | |
| *Sphk1* | 0.772 $\pm$ 0.158 | 2.418 $\pm$ 0.257 | **0.0079** | **0.0158** | 1.436 $\pm$ 0.141 | 0.580 $\pm$ 0.076 | **0.0007** | **0.0028** |
| *Sphk2* | 0.834 $\pm$ 0.094 | 1.898 $\pm$ 0.239 | **0.0159** | **0.0212** | 0.896 $\pm$ 0.074 | 0.996 $\pm$ 0.062 | 0.1209 | 0.1209 |
| S1P degrading enzymes | | | | | | | | |
| *Sgpl1* | 0.796 $\pm$ 0.063 | 1.810 $\pm$ 0.251 | **0.0044** | **0.0088** | 1.174 $\pm$0.021 | 0.846 $\pm$ 0.060 | **0.0009** | **0.0054** |
| *Sgpp1* | 0.714 $\pm$ 0.081 | 2.606 $\pm$ 0.429 | **0.0025** | **0.0075** | 0.904 $\pm$ 0.039 | 1.092 $\pm$ 0.056 | **0.0251** | **0.0301** |
| *Sgpp2* | 0.592 $\pm$ 0.267 | 2.872 $\pm$ 0.230 | **0.0079** | **0.0119** | 0.888 $\pm$ 0.094 | 1.204 $\pm$ 0.117 | 0.0692 | 0.0692 |
| S1P receptors | | | | | | | | |
| *S1pr1* | 0.854 $\pm$ 0.065 | 1.992 $\pm$ 0.320 | **0.0127** | **0.0222** | 0.900 $\pm$ 0.147 | 1.058 $\pm$ 0.170 | 0.1824 | 0.2128 |
| *S1pr2* | - | - | - | - | - | - | **-** | **-** |
| *S1pr3* | 0.860 $\pm$ 0.210 | 2.794 $\pm$ 0.356 | **0.0038** | **0.0133** | 1.352 $\pm$ 0.389 | 0.480 $\pm$ 0.068 | **0.0123** | **0.0222** |
| *S1pr4* | 0.860 $\pm$ 0.127 | 3.070 $\pm$ 0.149 | **0.0010** | **0.0070** | 1.286 $\pm$ 0.193 | 1.064 $\pm$ 0.306 | 0.1409 | 0.1973 |
| *S1pr5* | - | - | - |  | 0.943 $\pm$ 0.095 | 0.936 $\pm$ 0.084 | 0.9292 | 0.9292 |

**Suppl. Table 4. Regulation of endothelial gene expression by oxygen-glucose deprivation in murine and human brain endothelial cells.**

N = 9 for all timepoints. Data are presented as mean $\pm$ SD. *Adm – Adrenomedullin*, *Cdh5 – cadherin 5, Hmox1 – heme oxygenase 1,* *OGD – oxygen glucose deprivation, Pecam1 – Platelet Endothelial Cell Adhesion Molecule-1*, *S1P – sphingosine-1-phosphate, S1pr – S1P receptor, Sphk – sphingosine kinase, Sgpl – sphingosine lyase, Sgpp – sphingosine phosphatase, Vcam1 – Vascular Cell Adhesion Molecule 1, Vegfa – vascular endothelial growth factor A.*

| Target gene | **Human brain endothelial cells** | | | |
| --- | --- | --- | --- | --- |
|  | normoxia | hypoxia | P-value | adjusted P-value |
| Hypoxia-responsive genes | | | | |
| 6 hours OGD | | | | |
| *ADM* | 0.228 ± 0.021 | 2.610 ± 0.458 | **<0.0001** | **<0.000112** |
| *HMOX1* | 0.312 ± 0.143 | 1.794 ± 0.659 | **<0.0001** | **<0.000112** |
| *VEGFA* | 0.665 ± 0.108 | 1.544 ± 0.216 | **<0.0001** | **<0.000112** |
| 9 hours OGD | | | | |
| *ADM* | 0.401 ± 0.072 | 1.754 ± 0.329 | **<0.0001** | **<0.000112** |
| *HMOX1* | 0.436 ± 0.236 | 1.339 ± 0.456 | **<0.0001** | **<0.000112** |
| *VEGFA* | 0.588 ± 0.110 | 1.559 ± 0.174 | **<0.0001** | **<0.000112** |
| 16 hours OGD | | | | |
| *ADM* | 0.256 ± 0.086 | 1.812 ± 0.314 | **<0.0001** | **<0.000112** |
| *HMOX1* | 0.675 ± 0.539 | 0.864 ± 0.279 | 0.3498 | 0.3498 |
| *VEGFA* | 0.485 ± 0.179 | 1.595 ± 0.227 | **<0.0001** | **<0.000112** |
| S1P receptors | | | | |
| 6 hours OGD | | | | |
| *S1PR1* | 1.264 ± 0.109 | 0.748 ± 0.154 | **<0.0001** | **<0.00015** |
| *S1PR2* | 1.203 ± 0.126 | 0.769 ± 0.137 | **<0.0001** | **<0.00015** |
| *S1PR3* | 1.034 ± 0.119 | 0.853 ± 0.109 | **0.0007** | **0.00079** |
| 9 hours OGD | | | | |
| *S1PR1* | 1.323 ± 0.213 | 0.775 ± 0.181 | **<0.0001** | **<0.00015** |
| *S1PR2* | 1.230 ± 0.100 | 0.853 ± 0.060 | **<0.0001** | **<0.00015** |
| *S1PR3* | 1.082 ± 0.080 | 0.891 ± 0.072 | **0.0004** | **0.00051** |
| 16 hours OGD | | | | |
| *S1PR1* | 1.456 ± 0.313 | 0.699 ± 0.193 | **<0.0001** | **<0.00015** |
| *S1PR2* | 1.044 ± 0.090 | 1.042 ± 0.101 | 0.9623 | 0.9623 |
| *S1PR3* | 1.144 ± 0.099 | 0.876 ± 0.139 | **<0.0001** | **<0.00015** |
| Endothelial markers | | | | |
| 6 hours OGD | | | | |
| *PECAM1* | 1.045 ± 0.145 | 0.858 ± 0.065 | **0.0004** | **0.00051** |
| *CDH5* | 0.986 ± 0.075 | 0.960 ± 0.063 | 0.5987 | 0.5987 |
| *VCAM1* | 0.839 ± 0.364 | 1.729 ± 0.283 | **<0.0001** | **<0.00015** |
| 9 hours OGD | | | | |
| *PECAM1* | 1.232 ± 0.079 | 0.874 ± 0.099 | **<0.0001** | **<0.00015** |
| *CDH5* | 1.070 ± 0.089 | 0.815 ± 0.083 | **<0.0001** | **<0.00015** |
| *VCAM1* | 0.677 ± 0.162 | 1.643 ± 0.463 | **<0.0001** | **<0.00015** |
| 16 hours OGD | | | | |
| *PECAM1* | 1.329 ± 0.117 | 0.737 ± 0.101 | **<0.0001** | **<0.00015** |
| *CDH5* | 1.153 ± 0.095 | 0.707 ± 0.170 | **<0.0001** | **<0.00015** |
| *VCAM1* | 1.408 ± 0.237 | 0.832 ± 0.573 | **0.002** | **0.00225** |
| S1P generating enzymes | | | | |
| 6 hours OGD | | | | |
| *SPHK1* | 1.173 ± 0.133 | 0.974 ± 0.154 | **0.0009** | **0.00135** |
| *SPHK2* | 1.132 ± 0.042 | 0.839 ± 0.091 | **<0.0001** | **<0.0002** |
| 9 hours OGD | | | | |
| *SPHK1* | 1.034 ± 0.092 | 0.892 ± 0.035 | **0.0151** | **0.0151** |
| *SPHK2* | 1.190 ± 0.087 | 0.789 ± 0.110 | **<0.0001** | **<0.0002** |
| 16 hours OGD | | | | |
| *SPHK1* | 1.110 ± 0.139 | 0.950 ± 0.121 | **0.0065** | **0.0078** |
| *SPHK2* | 1.208 ± 0.146 | 0.924 ± 0.118 | **<0.0001** | **<0.0002** |
| S1P degrading enzymes | | | | |
| 6 hours OGD | | | | |
| *SGPL1* | 1.180 ± 0.124 | 0.780 ± 0.061 | **<0.0001** | **<0.0001** |
| *SGPP1* | 1.231 ± 0.118 | 0.741 ± 0.045 | **<0.0001** | **<0.0001** |
| 9 hours OGD | | | | |
| *SGPL1* | 1.090 ± 0.129 | 0.779 ± 0.095 | **<0.0001** | **<0.0001** |
| *SGPP1* | 1.160 ± 0.118 | 0.854 ± 0.027 | **<0.0001** | **<0.0001** |
| 16 hours OGD | | | | |
| *SGPL1* | 1.109 ± 0.110 | 0.705 ± 0.141 | **<0.0001** | **<0.0001** |
| *SGPP1* | 1.186 ± 0.137 | 0.911 ± 0.119 | **<0.0001** | **<0.0001** |

**Suppl. Table 5. Raw and Benjamini-Hochberg-adjusted P values for multi-gene expression analyses.**

Raw P-values obtained for target within each predefined experimental panel are shown together with the corresponding Benjamini-Hochberg false discovery rate-adjusted P-values. Multiple-comparison correction was applied within each experimental panel separately.

| **Target** | **raw P-value** | **adjusted P-value** |
| --- | --- | --- |
| **Figure 2** | | |
| SphK1 | 0.0109 | 0.0263 |
| SphK2 | 0.0272 | 0.0272 |
| Sgpl1 | 0.0181 | 0.0263 |
| Sgpp1 | 0.0210 | 0.0263 |
| Sgpp2 | 0.0031 | 0.0155 |
| **Figure 3** | | |
| S1pr1 | 0.0127 | 0.0169 |
| S1pr3 | 0.0038 | 0.0152 |
| S1pr4 | 0.0110 | 0.0169 |
| S1pr5 | 0.0312 | 0.0312 |
| **Figure 4** | | |
| Ocldn | 0.0625 | 0.0833 |
| Selp | 0.0016 | 0.0032 |
| Sele | 0.0012 | 0.0032 |
| Icam | 0.8929 | 0.8929 |
| **Suppl. Figure 2** | | |
| Sphk1 | 0.1250 | 0.1688 |
| Sgpl1 | 0.1477 | 0.1688 |
| Sgpp1 | 0.0625 | 0.1688 |
| S1pr1 | 0.1271 | 0.1688 |
| S1pr3 | 0.6775 | 0.6775 |
| Selp | 0.1477 | 0.1688 |
| Sele | 0.0625 | 0.1688 |
| Icam | 0.1164 | 0.1688 |
| **Suppl. Figure 3 a-e** | | |
| Sphk1 | 0.0350 | 0.0350 |
| Sphk2 | 0.0294 | 0.0350 |
| Sgpl1 | 0.0251 | 0.0350 |
| Sgpp1 | 0.0251 | 0.0350 |
| Sgpp2 | 0.0006 | 0.0030 |
| **Suppl. Figure 3 f-i** | | |
| S1pr1 | 0.0080 | 0.0160 |
| S1pr3 | 0.0308 | 0.0308 |
| S1pr4 | 0.0163 | 0.0217 |
| S1pr5 | 0.0004 | 0.0016 |
| **Suppl. Figure 5** | | |
| S1PR4 - 1d | 0.6238 | 0.9167 |
| S1PR4 - 3d | 0.9375 | 0.9375 |
| S1PR1- 1d | 0.6875 | 0.9167 |
| S1PR1 - 3d | 0.4309 | 0.9167 |
