# Supplementary material for: Circulating sphingosine-1-phosphate depletion is associated with endothelial activation and altered brain-endothelial S1P pathway expression in ischemic stroke
Source: Fluids Barriers CNS. 2026 Jun 12;23:82. doi: 10.1186/s12987-026-00828-z (PMC13285081; doi:10.1186/s12987-026-00828-z)
Supplement: Supplementary file 2 — Supplementary Material 2 [file 12987_2026_828_MOESM2_ESM.pdf]

# Figure 3

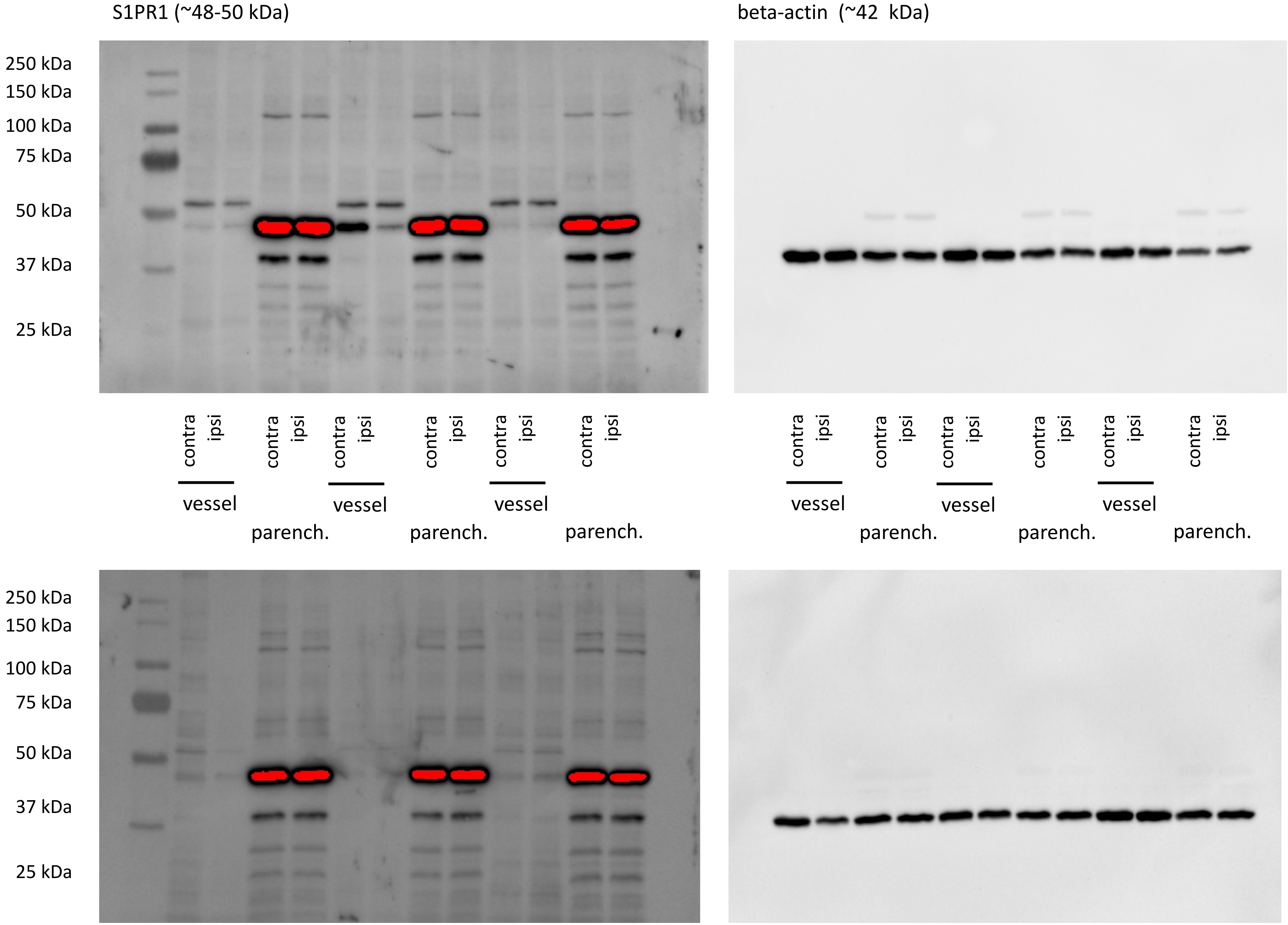
S1PR1 (~48-50 kDa)
beta-actin (~42 kDa)
250 kDa
150 kDa
100 kDa
75 kDa
50 kDa
37 kDa
25 kDa
contra
ipsi
vessel
parench.

# Figure 3

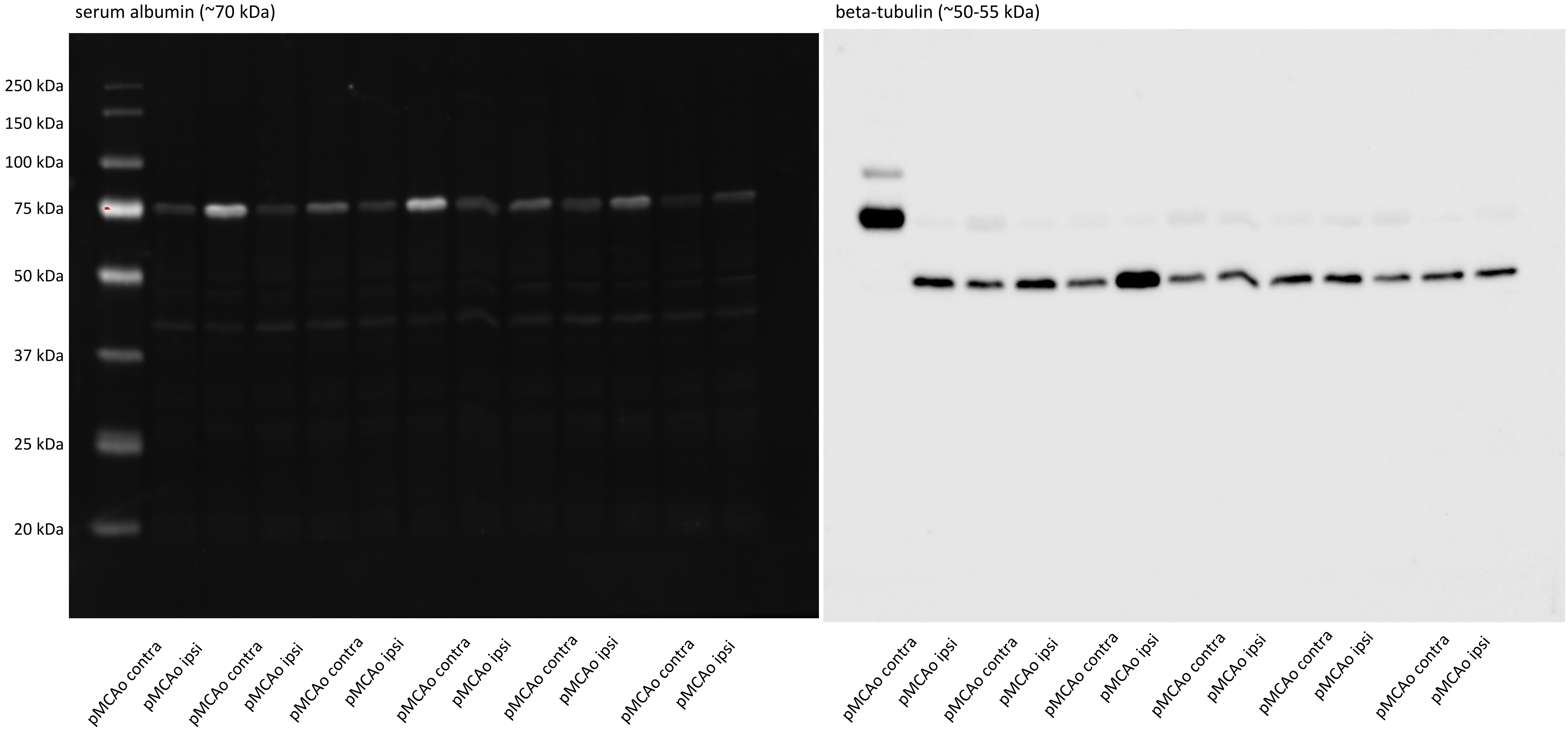

serum albumin (~70 kDa)
250 kDa
150 kDa
100 kDa
75 kDa
50 kDa
37 kDa
25 kDa
20 kDa
pMCAo contra
pMCAo ipsi
pMCAo contra
pMCAo ipsi
pMCAo contra
pMCAo ipsi
pMCAo contra
pMCAo ipsi
pMCAo contra
pMCAo ipsi
pMCAo contra
pMCAo ipsi
beta-tubulin (~50-55 kDa)
pMCAo contra
pMCAo ipsi
pMCAo contra
pMCAo ipsi
pMCAo contra
pMCAo ipsi
pMCAo contra
pMCAo ipsi
pMCAo contra
pMCAo ipsi
pMCAo contra
pMCAo ipsi

**Figure 4**

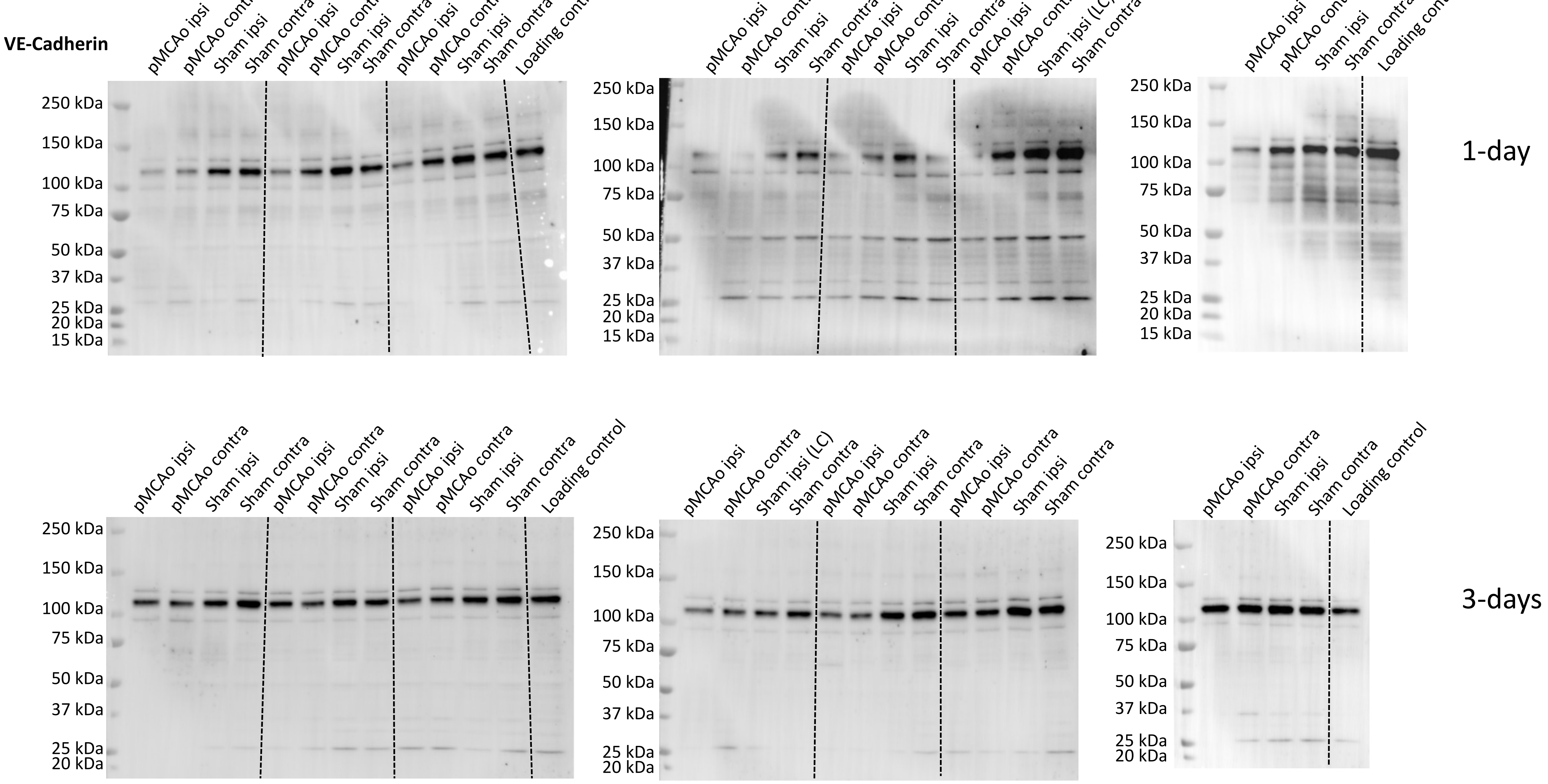
VE-Cadherin
pMCAo ipsi
pMCAo contra
Sham ipsi
Sham contra
Sham ipsi (LC)
Loading control
250 kDa
150 kDa
100 kDa
75 kDa
50 kDa
37 kDa
25 kDa
20 kDa
15 kDa
1-day
3-days

# Figure 4 / Suppl. Fig. 4

**Total protein**

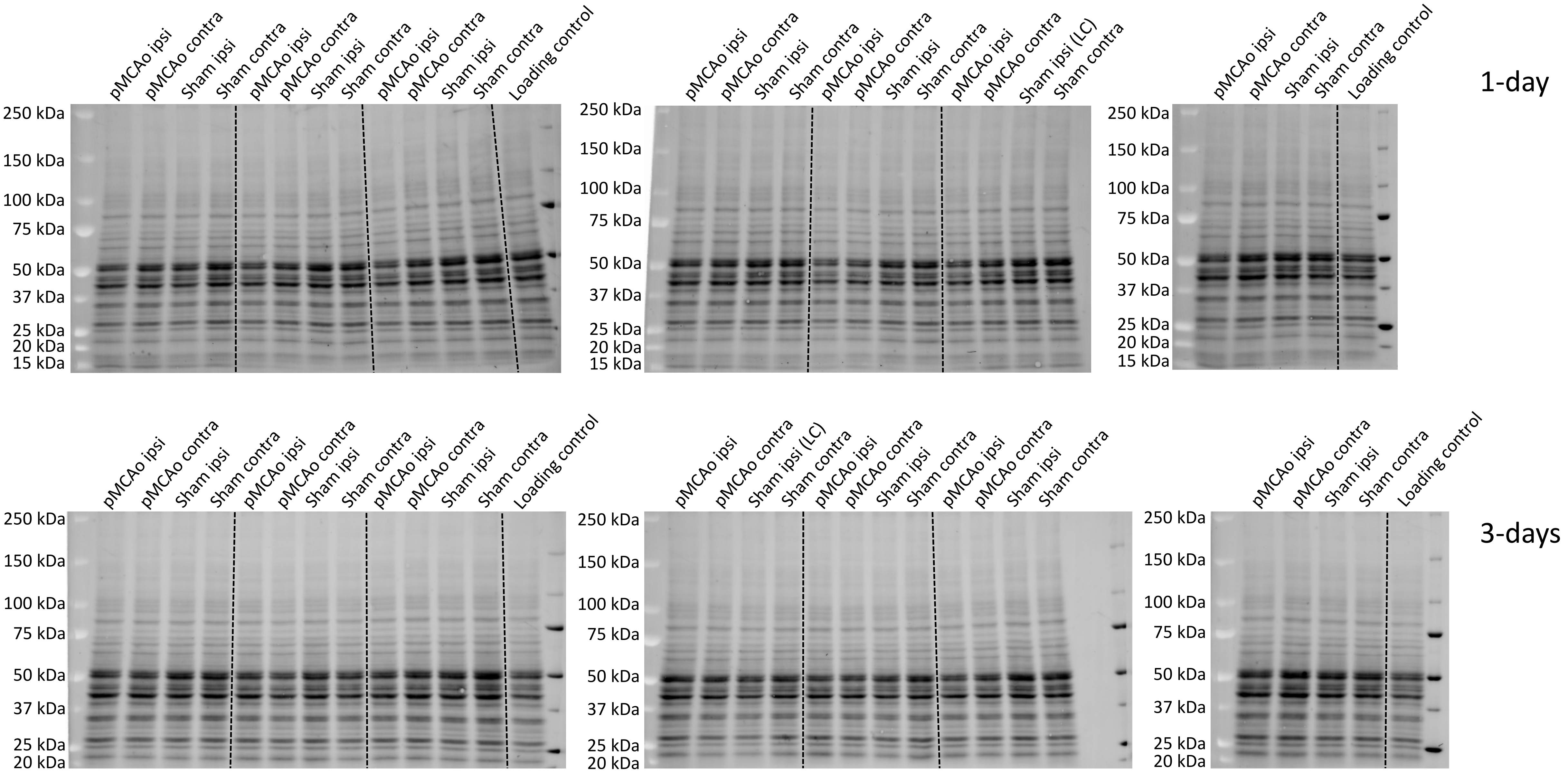

**Suppl. Fig. 4**

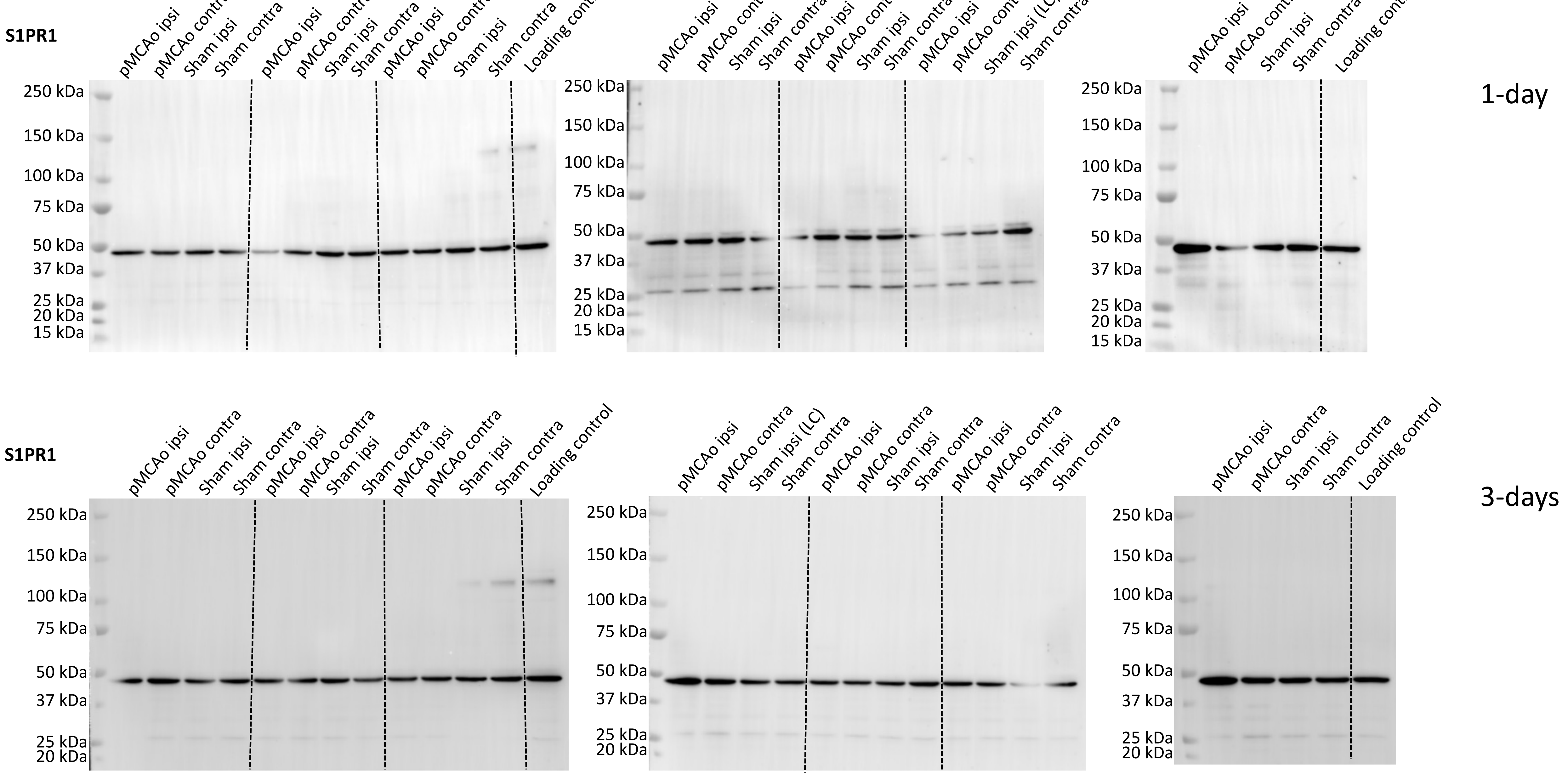
S1PR1
pMCAo ipsi
pMCAo contra
Sham ipsi
Sham contra
pMCAo ipsi
pMCAo contra
Sham ipsi
Sham contra
pMCAo ipsi
pMCAo contra
Sham ipsi
Sham contra
Loading control
250 kDa
150 kDa
100 kDa
75 kDa
50 kDa
37 kDa
25 kDa
20 kDa
15 kDa
pMCAo ipsi
pMCAo contra
Sham ipsi
Sham contra
pMCAo ipsi
pMCAo contra
Sham ipsi
Sham contra
pMCAo ipsi
pMCAo contra
Sham ipsi (LC)
Sham contra
250 kDa
150 kDa
100 kDa
75 kDa
50 kDa
37 kDa
25 kDa
20 kDa
15 kDa
pMCAo ipsi
pMCAo contra
Sham ipsi
Sham contra
Loading control
250 kDa
150 kDa
100 kDa
75 kDa
50 kDa
37 kDa
25 kDa
20 kDa
15 kDa
1-day
S1PR1
pMCAo ipsi
pMCAo contra
Sham ipsi
Sham contra
pMCAo ipsi
pMCAo contra
Sham ipsi
Sham contra
pMCAo ipsi
pMCAo contra
Sham ipsi
Sham contra
Loading control
250 kDa
150 kDa
100 kDa
75 kDa
50 kDa
37 kDa
25 kDa
20 kDa
pMCAo ipsi
pMCAo contra
Sham ipsi (LC)
Sham contra
pMCAo ipsi
pMCAo contra
Sham ipsi
Sham contra
pMCAo ipsi
pMCAo contra
Sham ipsi
Sham contra
250 kDa
150 kDa
100 kDa
75 kDa
50 kDa
37 kDa
25 kDa
20 kDa
pMCAo ipsi
pMCAo contra
Sham ipsi
Sham contra
Loading control
250 kDa
150 kDa
100 kDa
75 kDa
50 kDa
37 kDa
25 kDa
20 kDa
3-days

## Suppl. Fig. 4

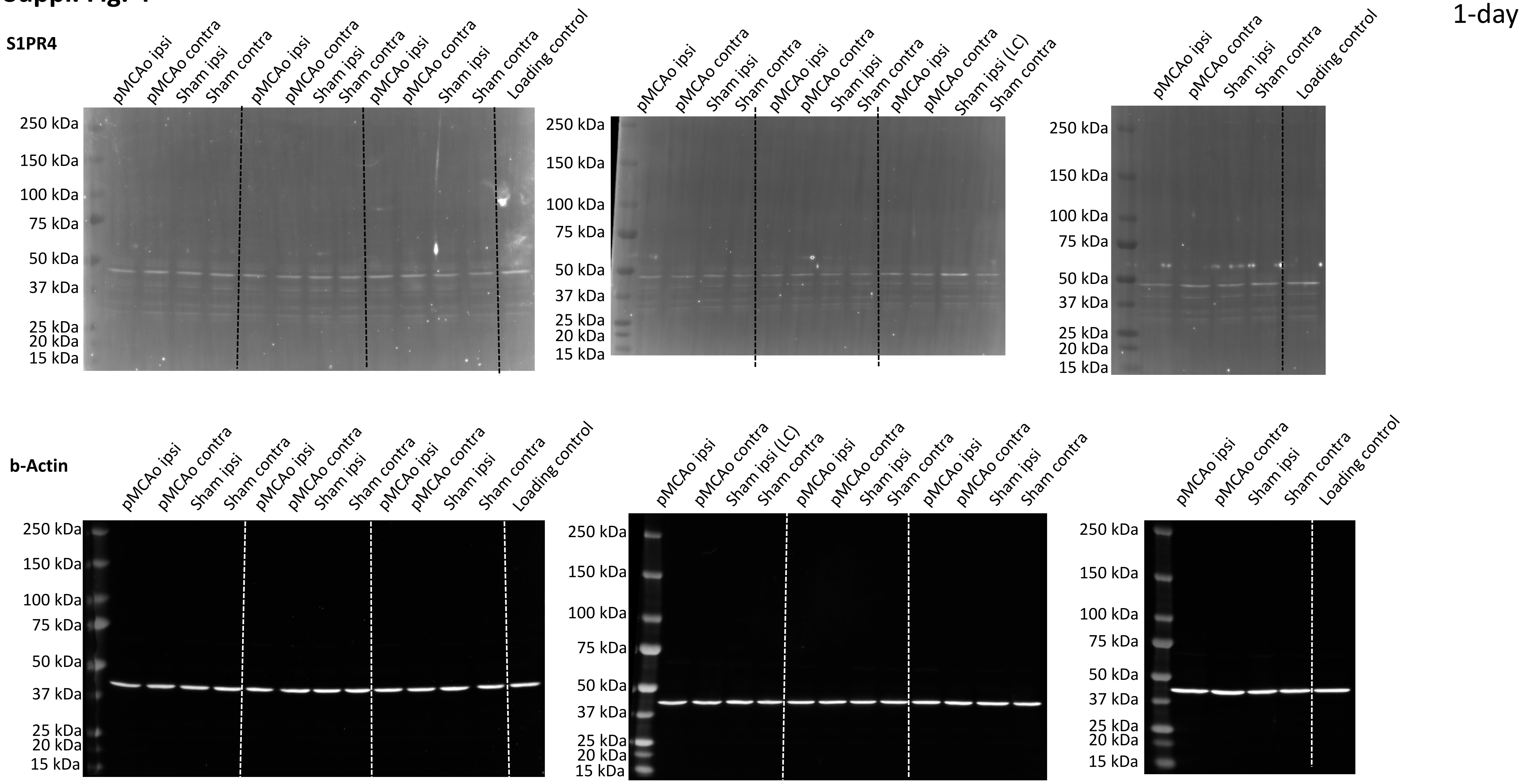
1-day
S1PR4
pMCAo ipsi
pMCAo contra
Sham ipsi
Sham contra
Loading control
Sham ipsi (LC)
250 kDa
150 kDa
100 kDa
75 kDa
50 kDa
37 kDa
25 kDa
20 kDa
15 kDa
b-Actin

**Suppl. Fig. 4**

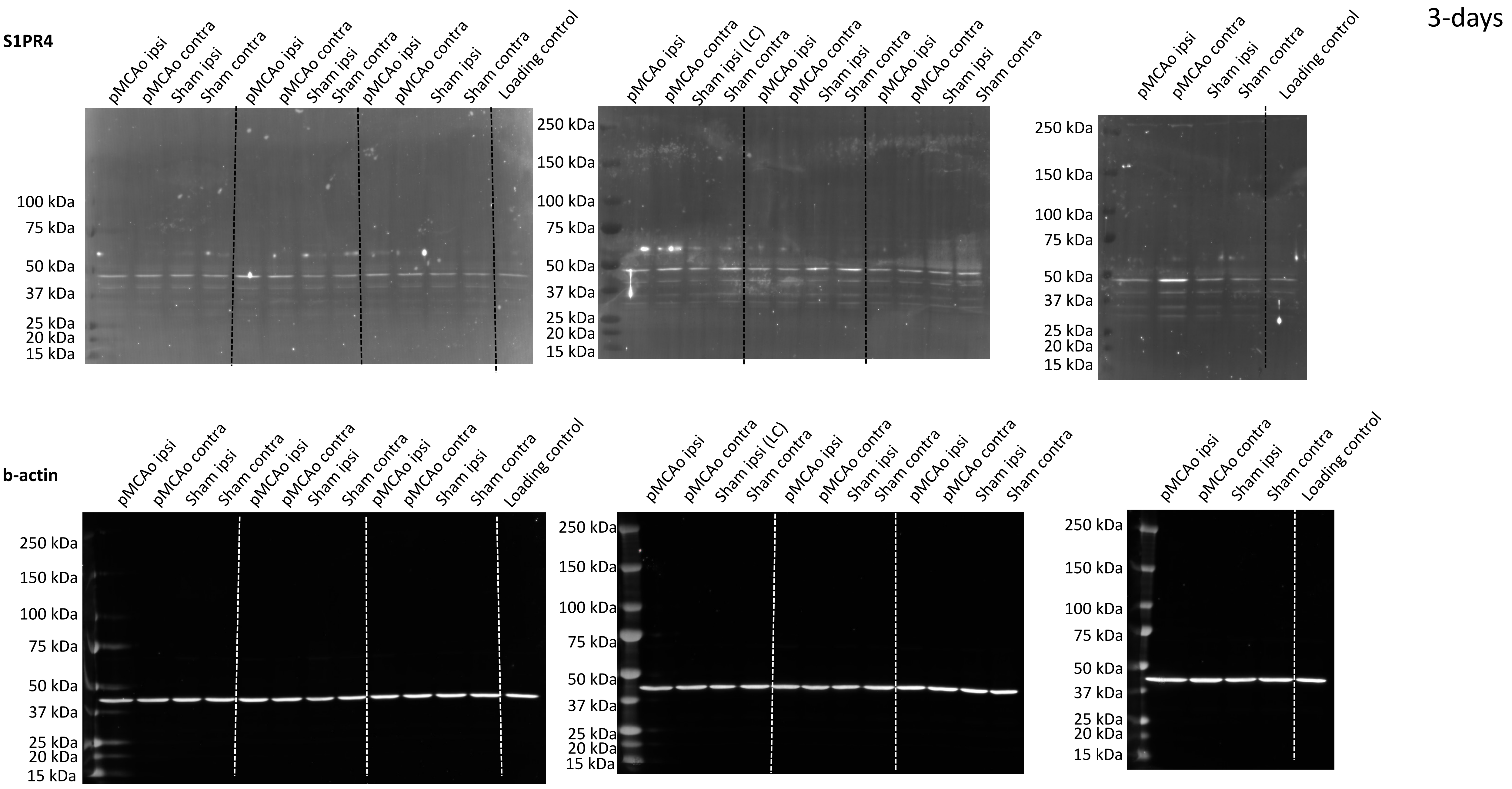
3-days
S1PR4
pMCAo ipsi
pMCAo contra
Sham ipsi
Sham contra
pMCAo ipsi
pMCAo contra
Sham ipsi
Sham contra
pMCAo ipsi
pMCAo contra
Sham ipsi
Sham contra
Loading control
100 kDa
75 kDa
50 kDa
37 kDa
25 kDa
20 kDa
15 kDa
pMCAo ipsi
pMCAo contra
Sham ipsi (LC)
Sham contra
pMCAo ipsi
pMCAo contra
Sham ipsi
Sham contra
pMCAo ipsi
pMCAo contra
Sham ipsi
Sham contra
250 kDa
150 kDa
100 kDa
75 kDa
50 kDa
37 kDa
25 kDa
20 kDa
15 kDa
pMCAo ipsi
pMCAo contra
Sham ipsi
Sham contra
Loading control
250 kDa
150 kDa
100 kDa
75 kDa
50 kDa
37 kDa
25 kDa
20 kDa
15 kDa
b-actin
pMCAo ipsi
pMCAo contra
Sham ipsi
Sham contra
pMCAo ipsi
pMCAo contra
Sham ipsi
Sham contra
pMCAo ipsi
pMCAo contra
Sham ipsi
Sham contra
Loading control
250 kDa
150 kDa
100 kDa
75 kDa
50 kDa
37 kDa
25 kDa
20 kDa
15 kDa
pMCAo ipsi
pMCAo contra
Sham ipsi (LC)
Sham contra
pMCAo ipsi
pMCAo contra
Sham ipsi
Sham contra
pMCAo ipsi
pMCAo contra
Sham ipsi
Sham contra
250 kDa
150 kDa
100 kDa
75 kDa
50 kDa
37 kDa
25 kDa
20 kDa
15 kDa
pMCAo ipsi
pMCAo contra
Sham ipsi
Sham contra
Loading control
250 kDa
150 kDa
100 kDa
75 kDa
50 kDa
37 kDa
25 kDa
20 kDa
15 kDa
